# Structural Basis of Teneurin-Latrophilin Interaction in Repulsive Guidance of Migrating Neurons

**DOI:** 10.1016/j.cell.2019.12.014

**Published:** 2020-01-23

**Authors:** Daniel del Toro, Maria A. Carrasquero-Ordaz, Amy Chu, Tobias Ruff, Meriam Shahin, Verity A. Jackson, Matthieu Chavent, Miguel Berbeira-Santana, Goenuel Seyit-Bremer, Sara Brignani, Rainer Kaufmann, Edward Lowe, Rüdiger Klein, Elena Seiradake

**Affiliations:** 1 Max Planck Institute of Neurobiology, Am Klopferspitz 18, Martinsried 82152, Germany; 2 Department of Biological Sciences, Institute of Neurosciences, IDIBAPS, CIBERNED, University of Barcelona, Barcelona, Spain; 3 Department of Biochemistry, Oxford University, Oxford OX1 3QU, UK; 4 Biological Integrative NMR Team, IPBS, Toulouse, France; 5 Center for Structural Systems Biology, University of Hamburg, Hamburg 22607, Germany; 6 Department of Physics, University of Hamburg, Hamburg 20355, Germany

**Keywords:** Teneurin, Latrophilin, FLRT, adhesion, repulsion, neuronal migration, cortex development, radial glia, pyramidal neuron

## Abstract

Teneurins are ancient metazoan cell adhesion receptors that control brain development and neuronal wiring in higher animals. The extracellular C terminus binds the adhesion GPCR Latrophilin, forming a *trans*-cellular complex with synaptogenic functions. However, Teneurins, Latrophilins, and FLRT proteins are also expressed during murine cortical cell migration at earlier developmental stages. Here, we present crystal structures of Teneurin-Latrophilin complexes that reveal how the lectin and olfactomedin domains of Latrophilin bind across a spiraling beta-barrel domain of Teneurin, the YD shell. We couple structure-based protein engineering to biophysical analysis, cell migration assays, and *in utero* electroporation experiments to probe the importance of the interaction in cortical neuron migration. We show that binding of Latrophilins to Teneurins and FLRTs directs the migration of neurons using a contact repulsion-dependent mechanism. The effect is observed with cell bodies and small neurites rather than their processes. The results exemplify how a structure-encoded synaptogenic protein complex is also used for repulsive cell guidance.

## Highlights

• Crystal structures reveal binding site for Latrophilin on the Teneurin YD shell
• A ternary Latrophilin-Teneurin-FLRT complex forms *in vitro* and *in vivo*
• Latrophilin controls cortical migration by binding to Teneurins and FLRTs
• Latrophilin elicits repulsion of cortical cell bodies/small neurites but not axons

## Graphical Abstract

**Figure:**
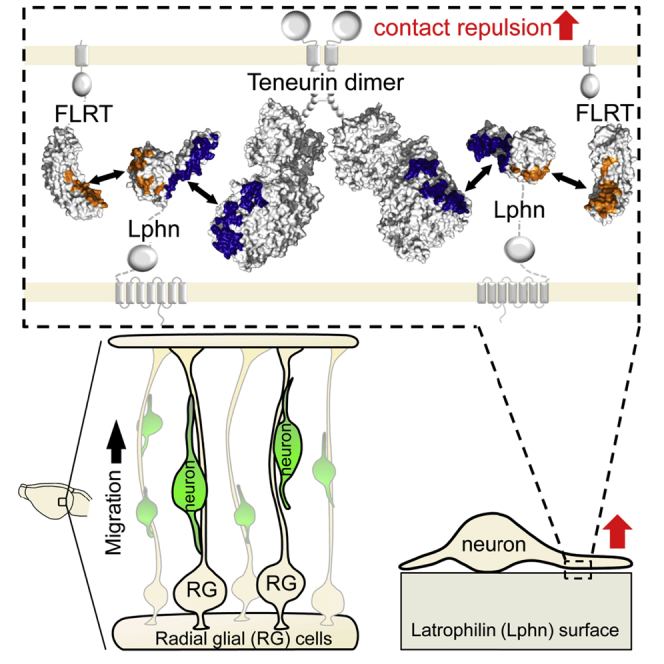

## Teaser

3D structures reveal how Latrophilin binds across the Teneurin protein and enable modeling of simultaneous FLRT binding. del Toro et al. show that this trimeric complex is important not only during synapse formation but also in earlier stages of development, where it guides the migration of young neurons by providing repulsive signals.

## Introduction

**Figure 1:**
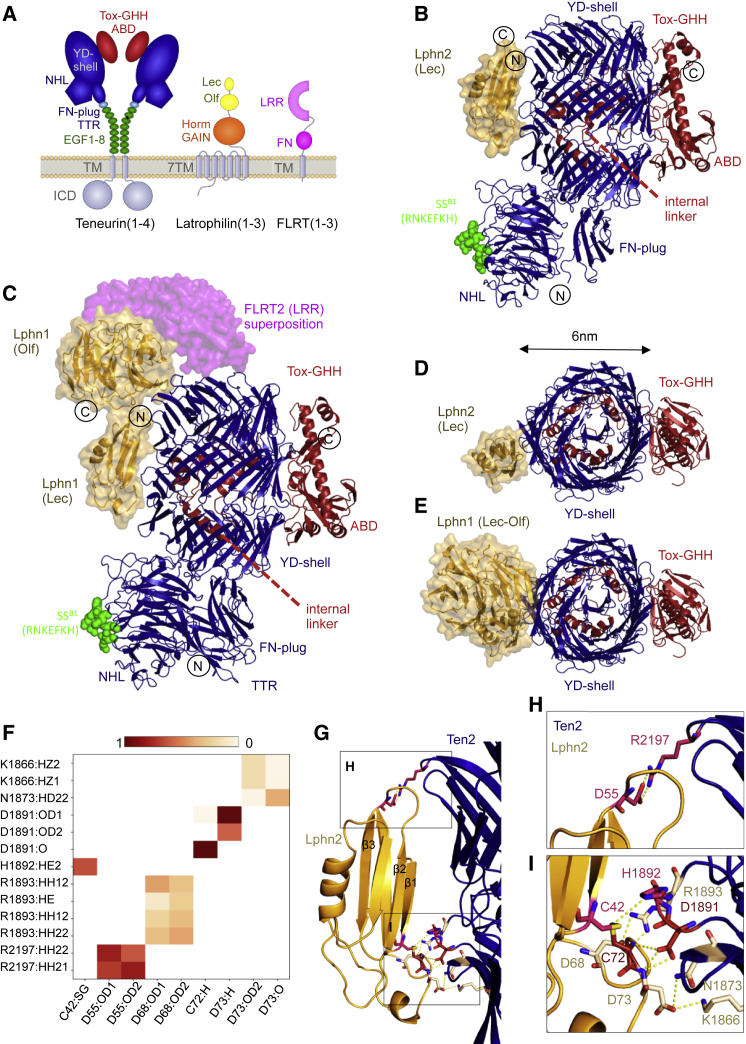
Crystal Structures of the Teneurin-Latrophilin Complex (A) Schematic of Teneurin, Latrophilin, and FLRT domain architectures. ABD, antibiotic-binding domain; EGF, epidermal growth factor domain; FN, fibronectin domain; FN plug, fibronectin plug domain; GAIN, GPCR autoproteolysis-inducing domain; Horm, hormone domain; ICD, intracellular domain; Lec, lectin domain; LRR, leucine-rich repeat; NHL, NCL-1, HT2A, and Lin-41 domain; Olf, olfactomedin domain; 7TM, seven-TM domain; TTR; transthyretin-like domain; TM, transmembrane helix; YD shell, tyrosine-aspartate repeats. (B) Crystal structure of the C-terminal domains of chicken Ten2 in complex with murine Lphn2 Lec. Colors are as in (A). N and C termini are indicated. The location of the alternatively spliced loop in the NHL domain (Berns et al., 2018) is indicated in green as spheres. (C) Crystal structure of the C-terminal domains of chicken Ten2 in complex with the murine Lphn1 Lec-Olf domains. The FLRT LRR domain (Jackson et al., 2016) was superposed by aligning the Olf domains of two structures. (D) Top view of the structure in (B). (E) Top view of the structure in (C). (F) Summary of the hydrogen bond analysis during a 50-ns restrained simulation. Atoms that contribute to stable hydrogen bonds between the two proteins are shown, and colored blocks indicate the stability of the bond during simulation. (G) The binding interface between Lphn Lec and the Teneurin YD shell comprises two main interacting areas (boxed areas). Selected hydrogen bonding residues are shown as sticks. Hydrogen bonds are shown as yellow lines. Interacting residues are colored according to the scheme in (G). (H and I) Magnified views of the two main binding areas as indicated in (G), top (H) and bottom (I).

Teneurins are eukaryotic cell adhesion receptors that are thought to have evolved through a horizontal gene transfer event, where fusion of a bacterial toxin gene to a eukaryotic receptor resulted in a large type II transmembrane protein (Tucker et al., 2012). They have been described in worms, insects, vertebrates, and single-celled choanoflagellates (Tucker et al., 2012). The four mammalian Teneurin homologs, Ten1 to Ten4, (also referred to as Ten-m1 to Ten-m4 and Odz-1 to Odz-4) are found predominantly, but not exclusively, in the nervous system, where they display complementary expression patterns from early development through to adulthood (Li et al., 2006, Ben-Zur et al., 2000, Kenzelmann et al., 2008, Zhou et al., 2003). In humans, Teneurins are risk loci in bipolar disorder (Croarkin et al., 2017, Green et al., 2013, Mühleisen et al., 2014, Psychiatric GWAS Consortium Bipolar Disorder Working Group, 2011), and schizophrenia (Ivorra et al., 2014), and Ten3 mutations have been implicated in microphthalmia (Aldahmesh et al., 2012) and general anosmia (Alkelai et al., 2016). The mammalian Teneurins are modular, ∼2,800-residue-long type II transmembrane proteins whose ∼250-kDa extracellular domain comprises at least 16 annotated domains (Figure 1A). We recently revealed the first X-ray crystallography and cryoelectron microscopy structures of an ∼200-kDa fraction of the *Gallus gallus* Ten2 and murine Ten3 extracellular domains. These revealed a conserved superfold of eight domains (Jackson et al., 2018). A similar cryoelectron microscopy structure of human Ten2 (Li et al., 2018) confirmed that key features of this fold are conserved. The structures revealed three signature motifs of the Teneurin fold: (1) the spiraling beta-barrel tyrosine-aspartate repeat “YD shell” domain, (2) a specialized “fibronectin plug” domain that seals off the YD shell at the N-terminal side, and (3) a beta-propeller referred to as the NCL-1, HT2A, and Lin-41 (NHL) domain. These three elements form a superfold that is widespread in bacterial genomes, suggesting that they represent an evolutionarily ancient uncharacterized family of secreted bacterial proteins (Jackson et al., 2018). The YD shell bears structural similarity to bacterial toxins of the TcB-TcC family (Busby et al., 2013, Gatsogiannis et al., 2013, Meusch et al., 2014). The regions upstream and downstream of the fibronectin (FN) plug, NHL, and YD shell are highly conserved in mammalian Teneurins. C-terminal of the YD shell is an ∼200-amino-acid linker that resides in the YD repeat shell and leads through the shell wall to form the antibiotic-binding-like (ABD) and Tox-GHH domains. The Tox-GHH harbors a colicin-like DNase fold (Ferralli et al., 2018, Jackson et al., 2018, Zhang et al., 2012) that includes a Teneurin C-terminal associated peptide, TCAP. This neuropeptide is either cleaved from the full Teneurin protein or transcribed separately and is thought to modulate murine stress behavior (Woelfle et al., 2016). Upstream of the Teneurin core fold lies the extracellular transthyretin (TTR)-like domain, a cysteine-rich region that is not structurally annotated, and eight epidermal growth factor (EGF) domains, two of which form disulphide bridges and mediate Teneurin dimer formation, presumably “*in cis*” (between Teneurins on the same cell). An ∼180-amino-acid-long linker leads from the EGF domains to the transmembrane (TM) helix and intracellular domain (ICD).

Pioneering studies in flies, which have two Teneurins, Ten-a and Ten-m, revealed roles in synaptic matching in the olfactory system (Hong et al., 2012) and at neuromuscular junctions (Mosca et al., 2012). In mice, Ten3 and Ten2 direct axonal wiring in the visual system (Antinucci et al., 2013, Antinucci et al., 2016, Dharmaratne et al., 2012, Leamey et al., 2007), the hippocampus (Berns et al., 2018), and of thalamostriatal projections (Tran et al., 2015). The NHL domain of mammalian Teneurins harbors an alternatively spliced loop (SS) (Berns et al., 2018) that determines homophilic binding of Teneurin in cell adhesion (Beckmann et al., 2013, Berns et al., 2018). Teneurins promote synapse development and *trans*-synaptic adhesion by also engaging in heterophilic interactions with Latrophilins (Boucard et al., 2014, Sando et al., 2019, Silva et al., 2011). Teneurin ectodomains that are proteolytically released from the cell surface bind to Latrophilins and act as attractants in axon guidance (Vysokov et al., 2018). Latrophilins (Lphn1–Lphn3 in mammals) are adhesion G-protein-coupled receptors (GPCRs) that have received attention because of their role as a calcium-independent receptor of latrotoxin (Davletov et al., 1996, Krasnoperov et al., 1996, Lelianova et al., 1997), their recently reported functions as mechanosensitive receptors (Liebscher and Schöneberg, 2016, Scholz et al., 2015, Scholz et al., 2017, Stoveken et al., 2015), and their roles in neurodevelopment disorders such as attention deficit hyperactivity disorder (ADHD) (Domené et al., 2011, Lange et al., 2012, van der Voet et al., 2016). Latrophilins also interact with FN leucine-rich repeat proteins (FLRTs) in synaptogenesis (O’Sullivan et al., 2012) and engage in large supercomplexes that include Uncoordinated-5 (Unc5) receptors (Jackson et al., 2015, Jackson et al., 2016, Seiradake et al., 2016). Latrophilins contain a short C-terminal ICD, a seven-TM GPCR domain (7TM), the extracellular GPCR autoproteolysis-inducing and hormone domains (GAIN/Horm), a glycosylated linker region (∼100 residues), and N-terminal olfactomedin (Olf) and lectin (Lec) domains (Figure 1A). FLRTs are single-spanning type I TM proteins with an extracellular leucine-rich repeat (LRR) domain that is connected to a downstream FN-like domain via a glycosylated linker (Figure 1A). The LRR domain of FLRT binds the Latrophilin Olf domain (Jackson et al., 2015, Lu et al., 2015, Ranaivoson et al., 2015), whereas Teneurins require the Latrophilin Lec domain for binding (Boucard et al., 2014). The linker between the Lec and Olf domains undergoes alternative splicing and determines the affinity of Latrophilin binding to Teneurin (Boucard et al., 2014). Recent work shows that FLRT3, Lphn2/3, and Ten2 work together as coincidence receptors in directing hippocampal synapse formation (Sando et al., 2019). How Teneurins and Latrophilins interact at the molecular level and whether Teneurin-Latrophilin interactions have functions in early neurodevelopment prior to wiring has remained unclear. Here we ask the following questions. What is the structural mechanism of the Teneurin-Latrophilin interaction? Is it compatible with the known FLRT-Latrophilin binding mechanism? What is the function of the synaptic proteins Teneurin, Latrophilin, and FLRT during early cortex development? With X-ray crystallography, we reveal the Latrophilin binding site on Teneurin at the lateral side of the YD shell domain. The Teneurin-Latrophilin binding mechanism we reveal is consistent with coincidence binding of FLRT to the Latrophilin Olf domain, and we suggest a ternary complex model using our previous structural data on the Latrophilin-FLRT interaction (Jackson et al., 2016). We use the structural results to probe for receptor functions in early cortical development and reveal a repulsion-mediated mechanism of cell body guidance. These results expand the functional repertoire of these receptors beyond a role in synapse development.

## Results

### The Latrophilin Lec Domain Binds across the Spiraling Beta-Barrel of the Teneurin YD Shell Domain

**Figure S1:**
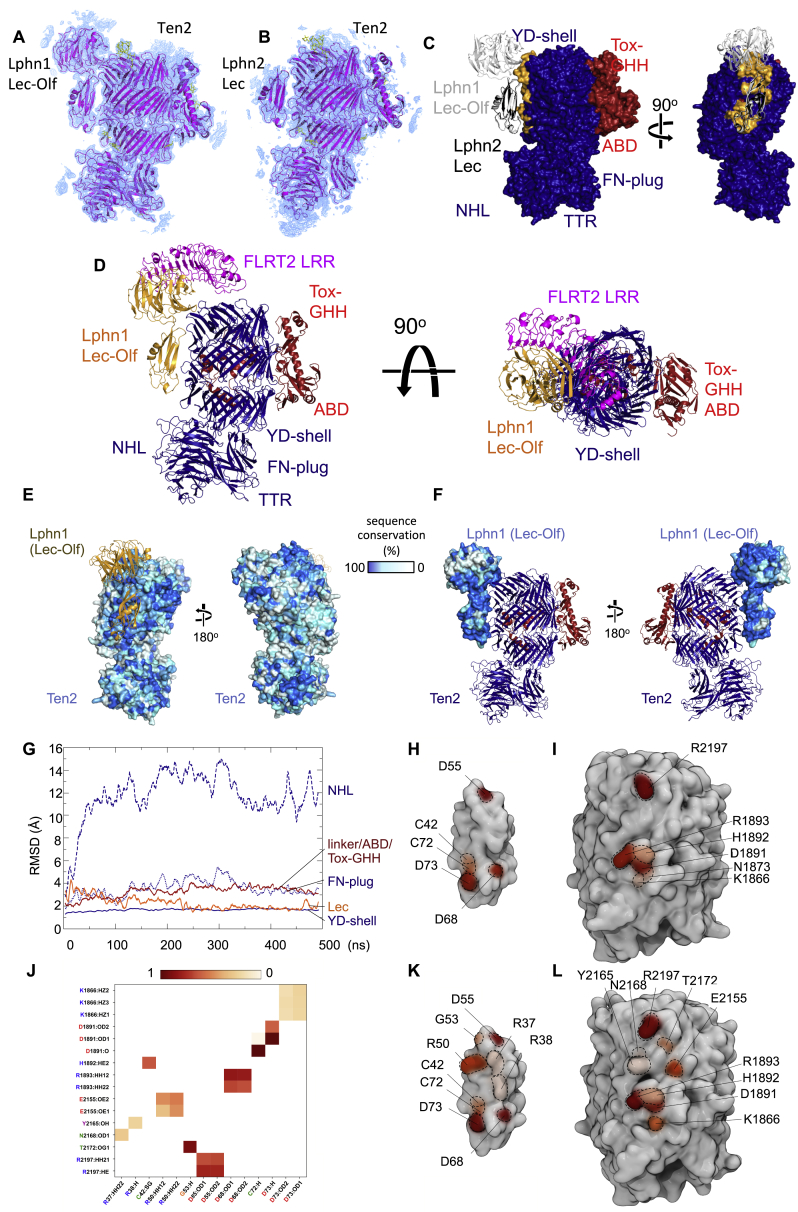
Crystal Structures of the Teneurin-Latrophilin Complex, Related to Figure 1 (A) The model of Ten2 in complex with Lphn1 Lec-Olf is shown in magenta, with modeled glycans shown in yellow. A bulk solvent-corrected 2Fo-Fc electron density map was calculated from the refined model and is shown in blue (1 σ-level), within a radius of 10 Å around the model. (B) As panel A, but showing one copy of the Ten2 - Lphn2 Lec complex and its 2FoFc map. (C) Superposition of Ten2 in complex with Lphn2 Lec (black ribbons) and Lphn1 Lec-Olf (white ribbons). Ten2 is shown in surface view and colored blue (TTR, FN-plug, NHL, YD-shell) and red (internal linker, ABD and Tox-GHH domains). The Teneurin residues that interface with Lphn1 Lec-Olf in the relevant complex structure are highlighted in yellow. (D) Superposition of FLRT2 LRR domain, as previously described when bound to Lphn3 Olf domain (Jackson et al., 2016) produces a model of the ternary complex as shown. (E) Ten2 surface models in complex with Lphn1 Lec-Olf (yellow ribbons) show conservation scores calculated with Consurf (Glaser et al., 2003) based on sequence conservation. The level of conservation is represented by color; Blue = highly conserved, white = not conserved. (F) As panel E, but showing the calculated surface conservation of Lphn1 (surface representation) and Ten2 as ribbons (N terminus: dark blue, C terminus: red). (G) A 500-ns (ns) simulation reveals the flexible movements of domains with respect to the YD-shell. Root mean square deviation (RMSD) values are plotted against time. Linker/ABD/Tox-GHH (2467-2797), YD-shell (1602-2466). NHL (237-1601), FN-plug (1047-1236). (H, I) Surface views of Ten2 and Lphn2 Lec complex as found in the crystal structure. Residues that contribute to stable hydrogen bonds in a 50 ns restrained simulation are highlighted in shades of red (see Figure 1F). (J) A summary of hydrogen-bond analysis during a 500 ns unrestrained MD simulation of the Ten2- Lphn2 Lec domain complex is shown. The colors are chosen to correlate with the stability of the bond during the simulation, ranging from red ( = stable) to white ( = not stable). Interacting residues from the 500 ns unrestrained simulation are mapped onto the surfaces the Lphn2 Lec domain (K) and Ten2 YD-shell (L).

Previous studies have demonstrated that the Lec domain of Latrophilin is essential and sufficient for binding to Teneurin proteins (Boucard et al., 2014). We produced murine Latrophilin 2 (Lphn2 Lec domain, residues 30–137) and chicken Teneurin 2 (residues 1,043–2,802) individually in HEK293 cells and mixed the purified proteins. Crystals of Ten2 and Lphn2 grew in sitting drops at 4°C. We collected X-ray diffraction data up to 3.6-Å resolution and solved the structure by molecular replacement (Figure 1B; PDB: 6SKE). Crystallographic details are summarized in Table S1. The data reveal the Lphn2 Lec binding site on the Teneurin YD shell, spanning the tiers of the spiraling YD shell domain (Figure 1B) and burying a total of ∼1,500 Å^2^ surface area. The Olf domain is thought to contribute to the binding between Teneurin and Latrophilin (Boucard et al., 2014). We therefore also expressed murine Latrophilin 1 (Lphn1 Lec-Olf domains, residues 29–395) in HEK293 cells and produced complex crystals with Ten2 (residues 955–2802) in sitting drops at 18°C. We collected X-ray diffraction data up to 3.86-Å resolution and solved the structure by molecular replacement (Figures 1C and S1A; PDB: 6SKA). This second structure confirms the conserved Lec binding site on the Ten2 YD shell domain (Figures 1B, S1B, and S1C). Most of the buried Teneurin surface is contacted by the Lec domain, which is consistent with previous reports showing that Lec is essential and sufficient for the interaction. The total buried surface in this complex is ∼2,500 Å^2^. In both structures, the C-terminal Tox-GHH/TCAP domain lies ∼6 nm away from the Latrophilin binding site (Figures 1D and 1E). FLRT is known to bind Latrophilin Olf via the concave surface of its LRR domain (Jackson et al., 2015, Lu et al., 2015). Superposition of the previously solved Lphn3-FLRT2 complex structure (Jackson et al., 2016) produces a model of how the three proteins likely interact in a ternary complex (Figures 1C and S1D). Sequence conservation in the binding interfaces of vertebrate Latrophilins and Teneurins (Figures S1E and S1F) suggests that the interaction has conserved functional importance that restrains sequence diversion in this area.

### Molecular Dynamics Simulations Reveal Two “Hotspots” on the Lphn2 Lec Domain

We chose the minimal complex of Lphn2 Lec-Ten2 (Figure 1B) for a set of molecular dynamics (MD) simulations to assess the stability of the complex and to better define the positions of side chains in the interface. A 500-ns unrestrained simulation of the complex showed little movement between the Lec and YD shell (root-mean-square deviation [RMSD], ∼0.2 nm), suggestive of a stable complex (Figure S1G). In contrast, the NHL domain drifted from its original position at the YD shell (RMSD, ∼1.2 nm), suggesting that it is connected via a flexible hinge. The FN plug domain, ABD, and Tox-GHH domains were displaced by ∼0.3 nm RMSD. These results are consistent with previous cryoelectron microscopy (cryo-EM) studies, which also suggested flexibility in these areas (Jackson et al., 2018, Li et al., 2018). We also performed a 50-ns simulation in which the protein backbone atom positions were restrained to refine the positions of the side chains. The results suggested two binding hotspots within the interface; one of these contacts is centered around D55, located in the loop between Lphn2 Lec strands β2 and β3 (Figures 1F–1H, and S1H). Analysis of the hydrogen bond pattern showed that D55 forms a stable salt bridge with R2197, located in the top tier of the Ten2 YD shell. A second contact is formed by the β1-β2 and β4-h1 loops of Lec, which are binding to the lower part of the Ten2 YD shell. Here, too, negatively charged residues (Lphn2 D68 and D73) bind a positive patch on Ten2 (R1893 and K1866) (Figures 1F, 1G, 1I, andS1I). Analysis of the hydrogen bonding patterns within the 500-ns unrestrained simulation revealed a similar pattern of hydrogen bonds (Figures S1J–S1L).

### Structure-Based Engineering in the Teneurin-Latrophilin Binding Sites

**Figure 2:**
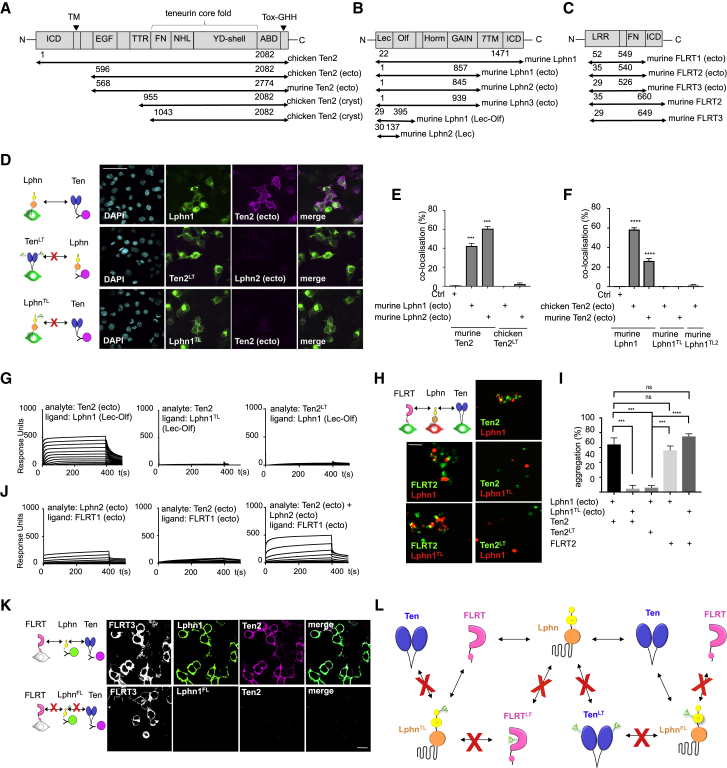
The Mutants Ten^LT^ and Lphn^TL^ Disrupt Teneurin-Latrophilin Binding (A–C) The Teneurin (Ten; A), Latrophilin (Lphn; B), and FLRT (C) constructs used in the study. (D) We tested receptor binding by expressing mVenus-tagged murine Lphn1 or chicken Ten2 (green) at the surface of HEK293 cells and detected the binding of His-tagged protein ectodomains (magenta) by immunofluorescence. DAPI labels cell nuclei (cyan). Representative images are shown. (E and F) Quantified results from the cell-based binding assays to test binding of surface expressed Ten with soluble Lphn (E), and surface expressed Lphn with soluble Ten (F). n = 12, ^∗∗∗∗^p < 0.0001, one-way ANOVA test with Tukey’s post hoc analysis. (G) In SPR experiments, we immobilized 220 response units of wild-type or mutant murine Lphn1 (Lec-Olf) on separate flow cells and injected Ten2 proteins using a 2-fold dilution series (highest concentration, 2.3 μM). Teneurin LT and Latrophilin TL mutants do not show binding. (H) K-562 cell aggregation assays show that the wild type, but not the mutants, promotes engagement of Latrophilin-expressing and Teneurin-expressing cells in *trans*. FLRT still interacts with Lphn^TL^. (I) Quantified results from the cell aggregation assay. n = 3; ^∗∗∗^p < 0.001, ^∗∗∗∗^p < 0.0001, one-way ANOVA test with Tukey’s post hoc analysis. (J) In SPR experiments, we immobilized 440 response units of FLRT1 (ecto) on separate flow cells and injected chicken Teneurin and murine Latrophilin analytes using the same concentration series in each experiment (highest concentration, 660 nM). Injecting both Teneurin and Latrophilin over FLRT1 gave an increased response. These results suggest that a ternary Teneurin-Lphn-FLRT complex forms *in vitro*. Results using FLRT2 and FLRT3 are shown in Figure S2. (K) We tested binding of His-tagged Lphn1 (green) and Ten2 (magenta) ectodomains to HEK293 cells expressing FLRT3 (white). Only wild-type Lphn1, not the FL mutant, forms a ternary complex with FLRT and Teneurin at the cell surface. (L) A diagram summarizing the binding capabilities of wild-type and mutant proteins. Scale bars represent 50 μm (D and I) and 20 μm (K).

**Figure S2:**
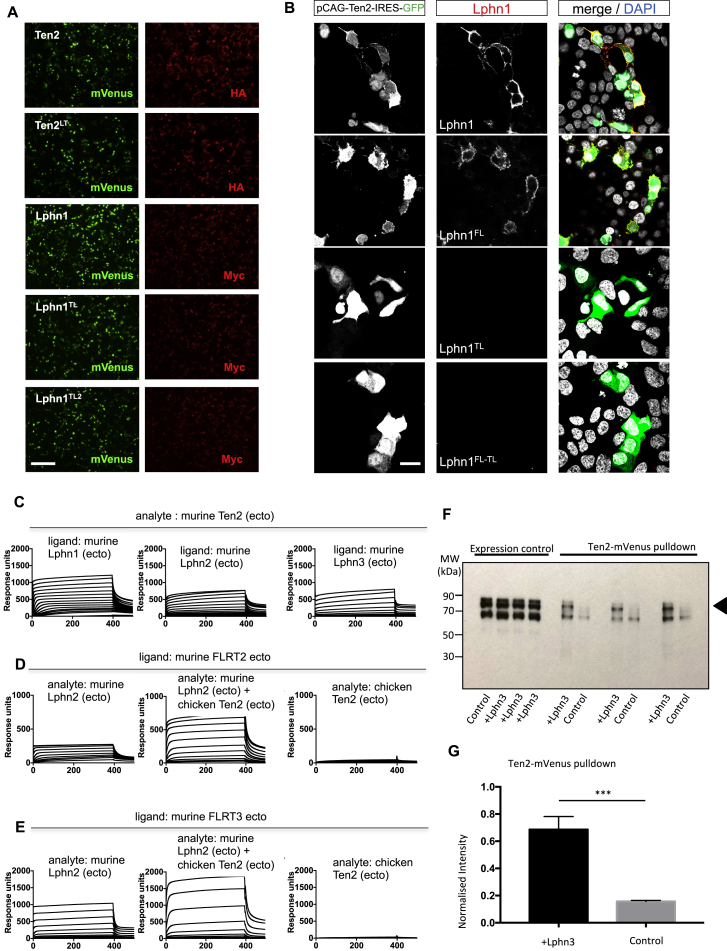
Teneurin, Latrophilin, and FLRT Interaction Studies, Related to Figure 2 (A) Teneurin and Latrophilin constructs were expressed in HEK293 cells with an intracellular mVenus and extracellular HA or Myc tag, respectively. We visualized cell surface expression with anti-HA or anti-Myc staining of fixed non-permeabilised cells. The staining shows that the constructs used in this study were all successfully expressed at the cell surface. Scale bar = 150 μm. (B) We tested the binding of Lphn1 (Lec-Olf) wild-type, single or double mutant proteins, clustered with anti-His and anti-mouse Alexa-594 (red), to HEK293 cells expressing Ten2 (green). Lphn1 and the non-FLRT binding (FL) mutant bind to Ten2. Non-Teneurin binding (TL) mutants do not bind. (C-E) SPR response curves are shown. The response units (y axis) are plotted against the time in seconds (x axis). (C) We immobilised 700 response units of murine Lphn1 (ecto), Lphn2 (ecto), or Lphn3 (ecto) on separate flow cells and injected a concentration series of mouse Teneurin 2 protein (highest concentration = 3.65 μM). (D, E) We immobilised 440, 300 and 1200 response units of FLRT2 (ecto) or FLRT3 (ecto). We injected Teneurin and Lphn proteins using the same concentration series in each experiment (highest concentration = 660 nM). (F) mVenus-tagged Ten2 was pulled down from HEK293T-cells co-transfected with FLAG-FLRT2, after incubating with either Lphn3-transfected cells or untransfected control cells. Anti-FLAG western blots show that FLRT2 is preferentially pulled down in the presence of Lphn3-expressing cells, compared to untransfected controls. Three representative repeats are shown. FLRT is highlighted by the arrow head. (G) Quantification of results shown in F. Results averaged from 6 experiments. Statistical significance was determined with a two-tailed unpaired t test where ^∗∗∗^p = 0.0003. Error bars show the s.e.m.

We produced a panel of Teneurin, Latrophilin, and FLRT constructs (Figures 2A–2C) and performed surface plasmon resonance (SPR) and cell-based binding experiments to validate our structural and simulation results. All relevant proteins were produced in mammalian HEK293 cells. We purified soluble ectodomains using affinity and size exclusion chromatography. TM constructs were tested for successful cell surface expression by immunostaining (Figure S2A; Jackson et al., 2015, Jackson et al., 2016, Seiradake et al., 2014). We also created “non-binding” mutants using an established method of introducing N-linked glycosylation sites at amino acid positions that are centrally located in the binding site. We refer to these mutants as “non-Latrophilin-binding Teneurin” (Ten^LT^) and “non-Teneurin-binding Latrophilin” (Lphn^TL^), in analogy to our previously published FLRT^LF^ mutants, the “non-Latrophilin-binding FLRT” proteins (Jackson et al., 2015). In addition, we produced a multiple-point mutant of Lphn1 that is based on our MD simulation results and does not depend on N-linked glycosylation: Lphn1^TL2^ (L39A, P51G, D54A, D67A, and D72A). A cell-based binding assay (Jackson et al., 2016), in which TM Teneurin or Latrophilin constructs are expressed on HEK293 cells and incubated with soluble Latrophilin or Teneurin ectodomains, showed that Ten2^LT^, Lphn1^TL^, and Lphn1^TL2^ have indeed lost their ability to interact (Figures 2D–2F), validating the crystal structures. We also tested the mutants using SPR with consistent results (Figure 2G). The non-FLRT binding Lphn^FL^ mutant still binds Teneurin (Figure S2B). To test the mutations in *trans*-cellular adhesion assays, we subjected them to a previously described cell aggregation assay where Teneurin-expressing cells adhere to Latrophilin-expressing cells (Berns et al., 2018). We show that Ten2^LT^ and Lphn1^TL^ do not promote K-562 cell adhesion (Figures 2H and 2I). The specificity of the mutants is given by the single point mutations that were used to generate them, and so they are fully functional otherwise. For example, Lphn1^TL^ cannot bind Teneurin, but it maintains its FLRT-binding capabilities. Therefore, Lphn1^TL^-expressing cells aggregate with FLRT2-expressing cells but not with Ten2-expressing cells (Figures 2H and 2I).

Our structural results suggest that a ternary complex of Teneurin-Latrophilin-FLRT could form via the described 1:1 binding surfaces of Lec-Ten2 and Olf-FLRT (Figure 1C). We tested the formation of such a ternary complex using SPR. Pre-mixed Latrophilin and Teneurin proteins produced an increased binding response to FLRT protein compared with Latrophilin alone. Ten2 binds all three Latrophilins (Lphn1–Lphn3; Figure S2C) but, on its own, does not bind FLRT (Figures 2J, S2D, and S2E). Therefore, this increase must be due to Teneurin interacting with FLRT via Latrophilin. A cell-based assay confirmed these binding results. We showed that Teneurin only binds to cell surface FLRT when wild-type Latrophilin is present and not when a non-binding Lphn mutant is used (Figure 2K). We also used a pull-down assay where cells expressing full-length FLRT and Teneurin were mixed with cells expressing full-length Latrophilin or with non-transfected control cells. Immunoprecipitation of Teneurin lead to efficient pull-down of FLRT when the cells were mixed with Latrophilin-expressing cells, but not when they were mixed with control cells (Figures S2F and S2G). These data confirm that the Latrophilin extracellular domain can bind both Teneurin and FLRT simultaneously *in vitro*. The findings are consistent with the coincident binding model of Latrophilin, Teneurin, and FLRT, proposed by others in synaptic development (Sando et al., 2019). A summary of the specific mutations we used here is given in Figure 2L.

### Teneurins and Latrophilins Are Expressed during Embryonic Cortical Development

**Figure 3:**
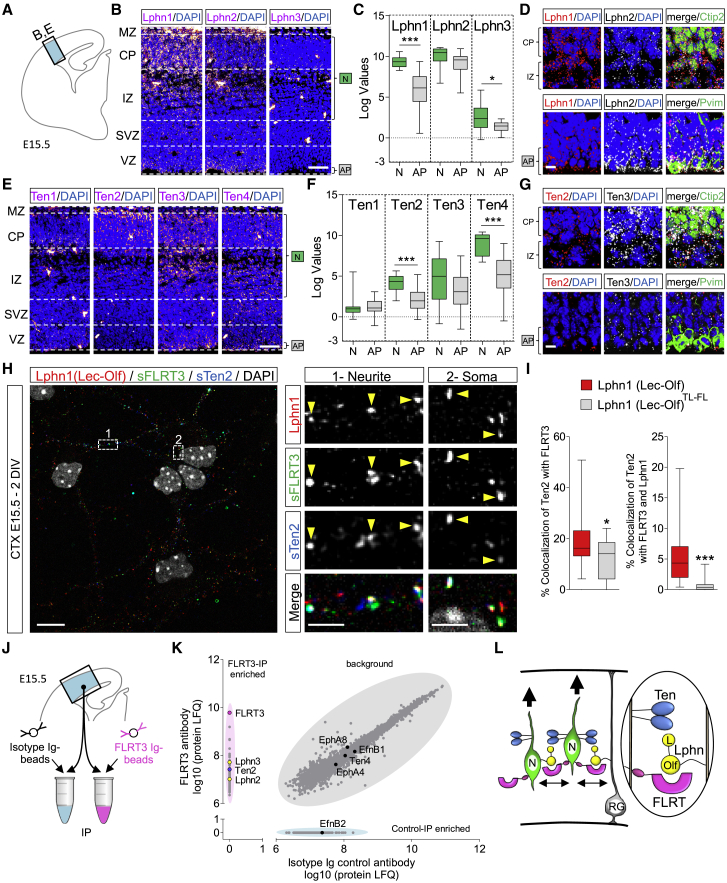
Teneurins and Latrophilins Are Expressed during Cortical Development (A) Scheme showing the location of the cortical region shown in (B) and (E). (B) *In situ* hybridization (ISH) for all Latrophilins (magenta) suggests that Lphn1 and Lphn2 are expressed in the cortex at E15.5. Nuclear staining with DAPI is shown in blue. The layers enriched in neurons (Ns) and apical progenitors (AP) are indicated. (C) Lphn1-3 expression in neurons and APs was quantified using published single-cell RNA profiling data (Kawaguchi et al., 2008; GEO: GSE10881). n = 15–20; ^∗^p < 0.05, ^∗∗∗^p < 0.001, two-tailed Student’s t test. The data are presented as whisker plots. (D) Double ISH for Lphn1 (red) and Lphn2 (white) combined with immunostaining for Pvim (green) or Ctip2 (green). The AP layer where radial glial (RG) cells are located, the cortical plate (CP), and the intermediate zone (IZ) are indicated. (E) ISH for Teneurins (magenta) reveals expression in neurons. Nuclear staining with DAPI is shown in blue. (F) Ten1-4 expression in neurons and APs was quantified using published data (Kawaguchi et al., 2008; GEO: GSE10881). n = 15–20; ^∗∗∗^p < 0.001, two-tailed Student’s t test. The data are presented as whisker plots. (G) Double ISH for Ten2 (red) and Ten3 (white) combined with immunostaining for the neuronal marker Ctip2 (green) and RG cell marker Pvim (green). The locations of the AP layer, CP, and IZ are indicated. (H) Surface staining for FLRT3 (red) and Ten2 (green) on E15.5 cortical neurons after 2 days *in vitro* (DIV), treated with Lphn1 (Lec-Olf) protein, shows that FLRT3 and Ten2 are expressed in both neurite (dashed rectangle labeled as 1) and soma compartments (dashed rectangle labeled as 2) and found in proximity to Lphn1 protein (high-magnification images on the right). For examples of super-resolution images, see Figure S3M. (I) Proximal Ten2 and FLRT3 staining was quantified for samples incubated with Lphn wild-type (H) or FL-TL mutant (Figure S3K) protein (graph on the left). We also quantified proximal staining for all three proteins (graph on the right). n > 30 fields from 3 experiments. ^∗^p < 0.001, ^∗∗∗^p < 0.001, two-tailed Student’s t test. (J) Scheme showing the location of the cortical region used for pull-down with control or FLRT3 antibodies. (K) Scatterplot results showing the clusters of proteins captured by control and FLRT3 antibody and revealed by mass spectrometry using label-free quantification (LFQ) quantitation. A pink ellipse delineates proteins enriched in FLRT3 pull-down. A blue ellipse indicates proteins enriched by control antibody. A gray ellipse shows proteins similarly enriched under both conditions. The results of two separate sets of pull-downs were averaged. Western blot results are shown in Figures S3P and S3Q. (L) A model showing that migrating neurons expressing Lphns, FLRTs, and Teneurins could interact in *trans* with Latrophilins located on radial glial fibers or neurons. The Lec and Olf domains of Latrophilins are indicated. Scale bars represent 150 μm (B and E), 15 μm (D, G, and H), and 2 μm (inset in H).

**Figure S3:**
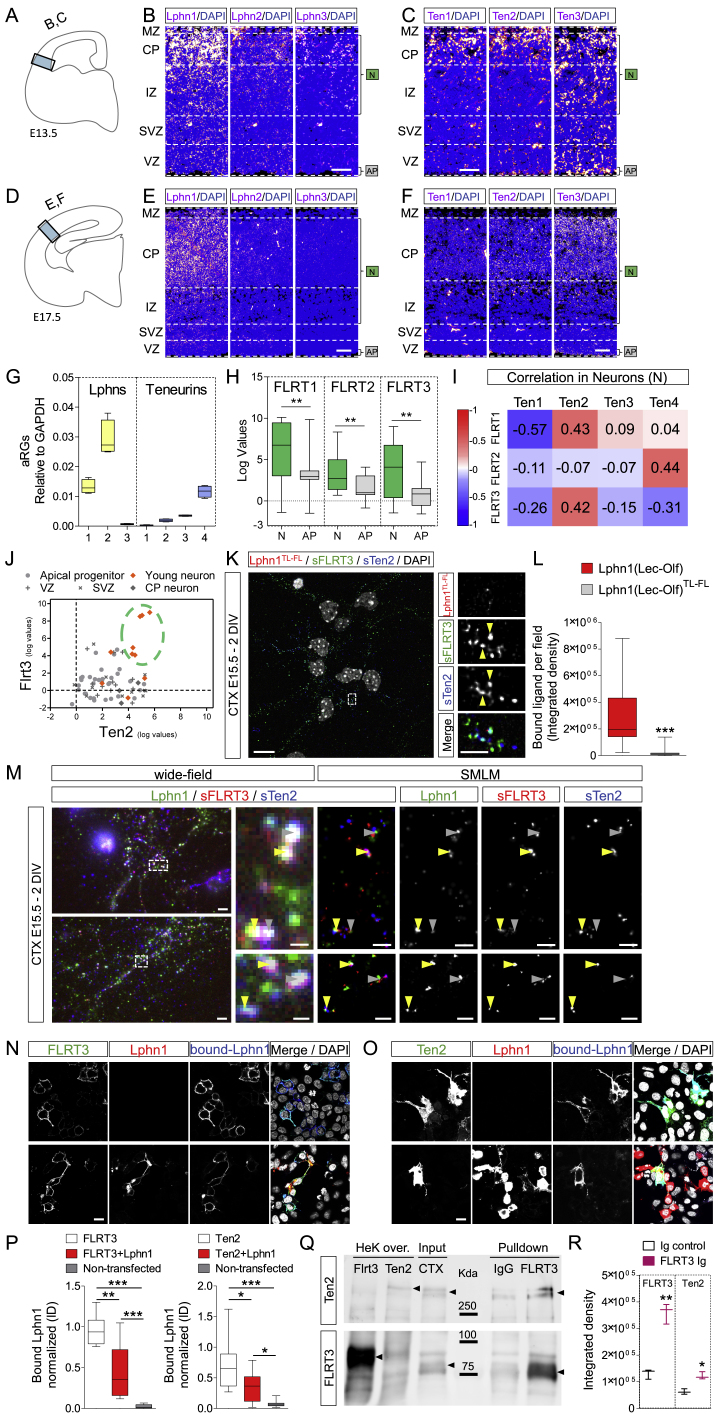
Teneurins, Latrophilins, and FLRTs Are Expressed during Cortical Development, Related to Figure 3 (A) Diagram showing the location of the coronal cortical region shown on panels B and C. (B and C) ISH for all Latrophilins (B) and Ten1,2 and 3 (C) colored in magenta show protein expression in the cortex of coronal sections of E13.5 mouse embryos. Nuclear staining with DAPI is shown in blue. (D) Diagram showing the location of the cortical region is shown on panels E and F. (E) ISH for all Latrophilins show that Lphn1 is expressed in the cortex of coronal sections of E17.5 mouse embryos. (F) ISH for Ten1, 2 and 3 show higher expression of Ten2 and 3 in the cortex of coronal sections of E17.5 mouse embryos. (G) Latrophilin and Teneurin expression levels normalized to GAPDH in mouse apical RG cells, using RNA profiling data published in Florio et al. (2015) (GSE65000). Lphn2 shows high expression levels compared to Lphn1 and Ten4. The data are presented as whisker plots. (H) FLRT1-3 expression in neurons (N) and apical progenitors (AP) using RNA profiling data published in Kawaguchi et al. (2008) (GEO: GSE10881). FLRT mRNA levels are high in neurons (N) compared to apical progenitors (AP). ^∗∗^p < 0.01, two-tailed Student’s t test. The data are presented as whisker plots. (I) Correlation analysis using RNA-Seq data for FLRTs and Teneurins in neurons, using data published in GEO: GSE10881. Ten2 expression correlates with FLRT1 and FLRT3, meaning it is present in the same neurons, while Ten4 expression shows correlation with FLRT2. (J) Scatter diagram showing data from 70 single cells from E14.5 mouse cortex, showing the variation in gene expression for FLRT3 and Ten2. The cluster of young neurons shows the strongest expression of both FLRT3 and Ten2. We used raw data previously published in GEO: GSE10881. (K) Surface staining for FLRT3 (red) and Ten2 (green) on E15.5 cortical neurons after 2 days of *in vitro* culture (DIV) treated with Lphn1^TL-FL^ (Lec-Olf) proteins for 20 min at room temperature. FLRT3 and Ten2 show some degree of co-localization (yellow arrowheads) (see quantification Figure 3I). The area in the dashed rectangle is magnified on the right. (L) Quantification of Lphn1 and the TL-FL double mutant binding to cortical neurons. The double mutant protein shows almost no binding to these cultures. n > 30 fields from 3 experiments. ^∗∗∗^p < 0.001, two-tailed Student’s t test. (M) Single Molecule Localization Microscopy (SMLM) imaging of E15.5 cortical neurons after 2 days *in vitro* (DIV), surface stained for surface FLRT3 (red) and surface Ten2 (blue) and treated with Lphn1 protein (green) for 20min at RT. Yellow arrowheads indicated co-localization within the SMLM resolution of approx. 30 nm. Gray arrowheads indicate signals that show co-localization in the conventional wide-field fluorescence, but only close proximity in SMLM. (N) Lphn1 (Lec-Olf), blue, binds to HEK293 cells expressing FLRT3 (green) in the presence or absence of Lphn1 *in cis* (red). Nuclei are stained with DAPI. (O) Lphn1 (Lec-Olf), blue, binds to HEK293 cells expressing Ten2 (green) in the presence of absence of Lphn1 *in cis* (red). Nuclei are stained with DAPI. (P) Quantification of data shown in panels (N) and (O). Lphn1 (Lec-Olf) binding was normalized with the intensity of the FLRT3 (N), Ten (O) or non-transfected signal. n > 10 fields. ^∗^p < 0.05, ^∗∗^p < 0.01, two-tailed Student’s t test. (Q) Control and FLRT3 pulldowns from mouse cortex E15.5 were analyzed by western blot. On the first two lanes we loaded 40μg of HEK cells overexpressing FLRT3 and Ten2, respectively, as a positive control. The third lane has 60μg of cortical lysate (CTX) input. The last two lanes are Ig control (left) and FLRT3 (right) pulldowns from 1mg CTX tissue. Ten2 and FLRT3 protein bands are indicated with black arrowheads. (R) Quantification of data shown in Q from 3 pull-down experiments (n = 3). ^∗^p < 0.05, ^∗∗^p < 0.01, two-tailed Student’s t test. Scale bar = 75 μm (B-F), 15 μm (K, N, O), 2 μm (inset of K, overview images in M), 500 nm (inset of M).

We previously showed that Latrophilin and FLRT proteins affect embryonic cortical neuron migration *in vitro* and *in vivo* (Jackson et al., 2015, Jackson et al., 2016, Seiradake et al., 2014, Del Toro et al., 2017, Yamagishi et al., 2011). Teneurins are expressed widely across the brain during development, including in the cortex (Kenzelmann et al., 2008, Rubin et al., 2002), where they could play a role in pathfinding (Vysokov et al., 2018). Here we asked whether Latrophilin-Teneurin binding regulates embryonic cortical migration. *In situ* hybridization (ISH) for Latrophilins revealed that Lphn1 and 2 are expressed in neuron-enriched layers (cortical plate [CP] and intermediate zone [IZ]) and the apical progenitor (AP)-enriched layer (ventricular zone [VZ]), where radial glial (RG) cell bodies are located, from embryonic days 13.5 [E13.5] to E17.5 (Figures 3A, 3B, S3A, S3B, S3D, and S3E). This finding is consistent with single-cell RNA profiling data from E14.5 mouse cortex (Kawaguchi et al., 2008) that also revealed expression of Lphn1 and Lphn2 in neurons and APs (Figure 3C). Combination of ISH with staining for the phosphorylated form of vimentin (Pvim), which labels dividing RGs, and the neuronal marker Ctip2 showed that Lphn1 and Lphn2 are expressed in neurons and RGs (Figure 3D). ISH for all four mouse Teneurins showed that these are predominantly expressed in neuron-enriched layers (CP and IZ) (Figure 3E; Figures S3C and S3F). In agreement with these results, analysis using data from two RNA-seq databases showed that Ten2 and Ten4 are highly expressed in neurons compared with APs (Figure 3F; Kawaguchi et al., 2008). Some Ten4 expression is also detected in RGs (Figure S3G; Florio et al., 2015). These results were further confirmed by co-staining with the neuronal marker Ctip2 and the RG marker Pvim. Ten2 and Ten3 showed reduced staining in APs compared with neuron-enriched layers (Figure 3G).

Given that FLRTs are also enriched in neurons during cortical development (Seiradake et al., 2014; del Toro et al., 2017; Figure S3H), we asked whether FLRTs and Teneurins are co-expressed in migrating cortical neurons. Single-cell RNA profiling analysis (Kawaguchi et al., 2008) showed a positive correlation between Ten2 and FLRT1/3 and Ten4 with FLRT2 in cortical neurons (Figure S3I). Co-expression of Ten2 and FLRT3 was strongest in migrating neurons compared with other cell types (Figure S3J). Surface staining for both Ten2 and FLRT3 revealed that both receptors are located in close proximity on the cell body and neurites/growth cones of cortical neurons. Addition of Lphn1 (Lec-Olf) protein induced more proximal localization of FLRT3 and Ten2 compared with addition of the Lphn1 (Lec-Olf) non-Teneurin and non-FLRT-binding (TL-FL) mutant protein. The mutant protein also bound less to cultured neurons compared with the wild type, and it co-localized less with Ten2 and FLRT3 staining (Figures 3H, 3I, and S3K–S3M). Latrophilin expression *in cis* can moderate but does not abolish Teneurin or FLRT binding to externally presented Latrophilin (Figures S3N–S3P).

In agreement with these results, our crystallography, SPR, and cell-binding experiments suggest that Latrophilin, FLRT, and Teneurin physically interact (Figures 1 and 2). Pull-down experiments using E15.5 mouse brain lysate show that Ten2 and Latrophilins co-immunoprecipitate with FLRT, suggesting that the three proteins also interact *in vivo* (Figures 3J, 3K, S3Q, and S3R). Based on these results, we developed a working model in which migrating neurons co-expressing Lphns, Teneurins, and FLRTs interact with Lphns present in RG cells and/or other cortical neurons (Figure 3L). We proceeded with functional analysis to understand the roles of these proteins in early cortical development.

### Latrophilin Regulates Cell Migration via Teneurins and FLRTs

**Figure 4:**
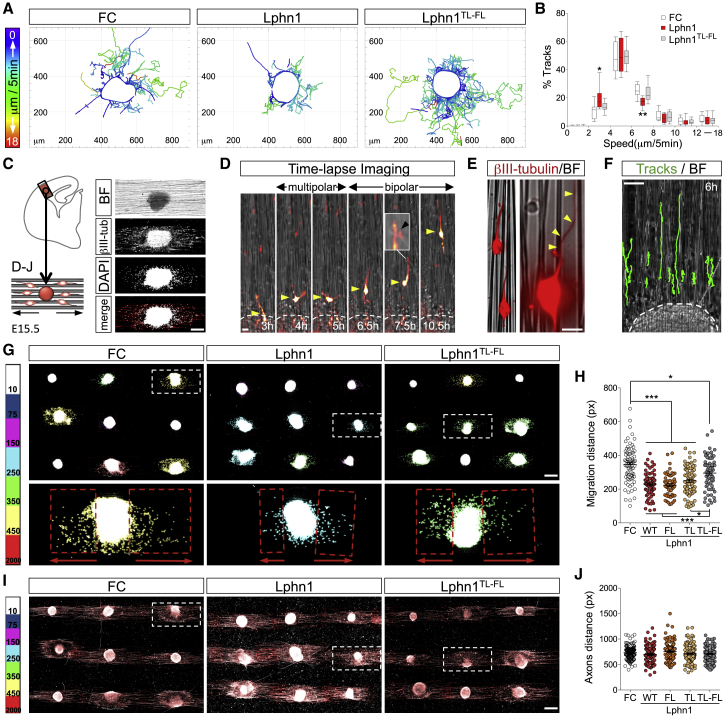
Latrophilin Interaction with Teneurin and FLRT Slows Down Cell Migration *In Vitro* (A) Time-lapse analysis of cortical neurons exiting E15.5 cortical explants on surfaces coated with FC (control), Lphn1 (Lec-Olf), or Lphn1^TL-FL^ (Lec-Olf) proteins. Neurons were tracked (lines) and colored based on the speed of migration. (B) Average speed frequency distributions. n > 10 movies per condition. ^∗^p < 0.05, ^∗∗^p < 0.01, one-way ANOVA test with Tukey’s post hoc analysis. (C) Diagram depicting the nanofiber assay. (D) Snapshots from a time-lapse video of an mCherry-expressing neuron (red) migrating on nanofibers coated with FC control protein. A yellow arrowhead points to the cell soma. The transition from multipolar to bipolar morphology is visible. The leading process showed occasional branching (magnification, black arrowhead). (E) Cortical neurons stained with β-III-tubulin (red) with bipolar morphology (left) and an example of a leading process switching to a neighboring fiber (right, yellow arrowhead). (F) Tracks (green) from time-lapse imaging of neurons migrating on nanofibers. (G) Explants growing on nanofibers coated with FC, Lphn1, and Lphn1^TL-FL^ Lec-Olf for 2 DIV. DAPI staining is color-coded based on the average distance from the explant, indicating length of migration (see also Figure S4E). (H) Quantification of the data shown in (G). n = 144 explants (36 explants per experiment, 4 experiments per condition). See full images in Figure S4F. ^∗^p < 0.05, ^∗∗^p < 0.01, ^∗∗∗^p < 0.001, one-way ANOVA test with Tukey’s post hoc analysis. (I) The same explants as shown in (G), stained using β-III-tubulin. (J) Quantification of the data shown in (I). n = 144 explants (36 explants per experiment, 4 experiments per condition) as described in (H). Scale bars represent 150 μm (C), 15 μm (D and E), and 250 μm (F, G, and I).

**Figure S4:**
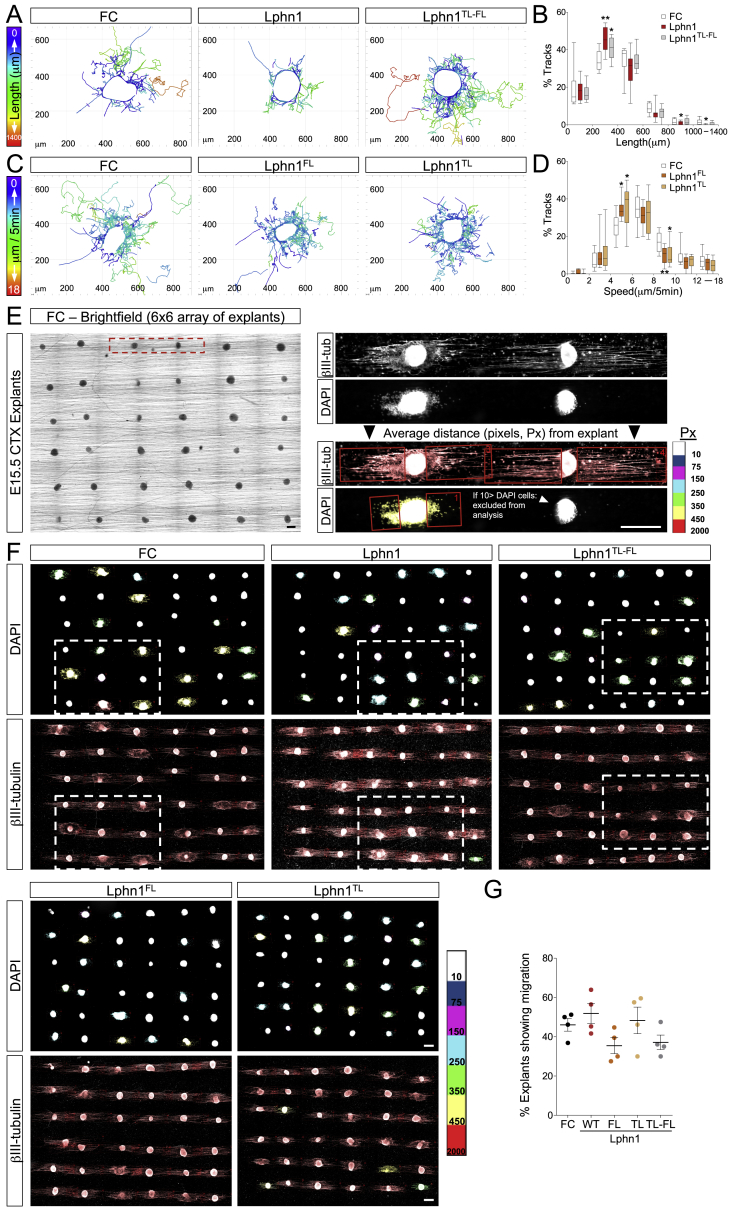
Latrophilin Interaction with Teneurin and FLRT Slows Down Cell Migration *In Vitro*, Related to Figure 4 (A) Time-lapse analysis of cortical neurons migrating from E15.5 cortical explants on surfaces coated with FC (control), Lphn1 or Lphn1^TL-FL^ proteins. Neurons were tracked (colored lines) and color-coded based on length of individual tracks. (B) Average length frequency distribution of all tracks in all conditions. n > 10 movies per condition. ^∗^p < 0.05 and ^∗∗^p < 0.01, one-way ANOVA test with Tukey’s post hoc analysis. The data are presented as whisker plots. (C) Time-lapse analysis of cortical neurons migrating from E15.5 cortical explants on surfaces coated with FC (control), murine Lphn1^FL^ (Lec-Olf) or Lphn1^TL^ (Lec-Olf) proteins. Neurons were tracked (colored lines) and color-coded based on speeds in individual tracks. (D) Average speed frequency distribution of all tracked neurons in all conditions. n > 10 movies per condition.^∗^p < 0.05 and ^∗∗^p < 0.01, one-way ANOVA test with Tukey’s post hoc analysis. The data are presented as whisker plots. (E) Scheme illustrating the organization of the 36 explants that are cultured per condition in each experiment and condition. The higher magnification of 2 explants shown on the right, shows growth and extension of axons after 2 days (DIV) (βIII-tubulin staining). DAPI staining illustrates the presence of explants that show migration of neurons (left explant, defined as explants showing more than 10 DAPI cells) while others only show extension of axons (right explant). Explants that show migration are quantified by drawing a rectangle on both sides containing the DAPI cells, based on the averaged distance from both sides, the explant is color-coded following the scale bar on the right. Same quantification applies for the extension of the axons. (F) Full images from one experiment with all conditions (control FC, Lphn1 and its mutant versions Lphn1^FL^, Lphn1^TL^ and Lphn1^TL-FL^). Axon extension is not affected in any of these conditions, and occurs in all explants (βIII-tubulin). In contrast, DAPI staining reveals that only 40%–60% of the explants show exiting cell migration (defined as more than 10 DAPI+ cells exiting the explants). (G) Quantification of the percentage of explants with cell migration shown in (F). n = 4 experiments per condition as described in (E). Scale bar = 300 μm (E and F).

To study the effects of Latrophilin-Teneurin binding on cortical neuron migration, we performed time-lapse imaging of E15.5 embryonic cortical explants grown on Lphn1 (Lec-Olf)-coated dishes and measured the migration of neurons exiting the explant (Figures 4A and S4A). Using automatic tracking (Video S1), we found that cortical neurons migrated slower and shorter distances on Lphn1 (Lec-Olf)-coated surfaces compared with control surfaces (Figures 4B and S4B). Similar effects were observed with the TL-FL Lec-Olf single mutants (Figures S4C and S4D; Video S1). In contrast, neurons migrating on the double-mutant protein (Lphn^TL-FL^ Lec-Olf) behaved similarly as those on control protein.

Video S1. Time-Lapse Analysis of Cortical Neurons Migrating from E15.5 Cortical Explants on Surfaces Coated with FC (Control), Lphn1, or Lphn1^TL-FL^ Proteins and Time-Lapse Analysis of Cortical Neurons Migrating from E15.5 Cortical Explants on Surfaces Coated with FC (Control), Lphn1^TL^, or Lphn1^FL^ Proteins, Related to Figure 4 First movie: Time-lapse analysis of cortical neurons migrating from E15.5 cortical explants on surfaces coated with FC (control), Lphn1, or Lphn1^TL-FL^ proteins. We coated surfaces with FC (control), Lphn1 or Lphn1^TL-FL^ proteins by adding 50μg/ml of these proteins in PBS on 60mm delta surface dishes (Thermofisher). After 30min incubation at 37°C, the dishes were washed three times with PBS and coated with 20μg/ml laminin for ∼2 hours at 37°C. Cortical explants from E15.5 mouse embryos were cultured in neurobasal, supplemented with B27 and 0.4% methylcellulose. After 4 hours in culture, the explants were imaged with a Zeiss Axiovert 200M microscope equipped with a temperature-controlled carbon dioxide incubation chamber set to 37°C, 65% humidity and 5% CO_2_. Illumination was provided by an X-Cite lamp (series 120, Lumen Dynamics Group), and images were recorded by a Coolsnap HQ camera (Photometrics). Sequential images were acquired every 5 min. Analysis was carried out using Imaris v9 (Bitplane), all cells were tracked and the averaged speed and track length analyzed. The brightfield frames are shown, with the identified tracks, color-coded based on average speed, shown below. Second movie: Time-lapse analysis of cortical neurons migrating from E15.5 cortical explants on surfaces coated with FC (control), Lphn1^TL^ or Lphn1^FL^ proteins. We coated surfaces with FC (control), Lphn1^TL^ or Lphn1^FL^ proteins by adding 50μg/ml of these proteins in PBS on 60mm delta surface dishes (Thermofisher). After 30 min incubation at 37°C, the dishes were washed three times with PBS and coated with 20μg/ml of laminin for ∼2 hours at 37°C. Cortical explants from E15.5 mouse embryos were cultured in neurobasal, supplemented with B27 and 0.4% methylcellulose. After 4 hours in culture, the explants were imaged with a Zeiss Axiovert 200M microscope equipped with a temperature-controlled carbon dioxide incubation chamber set to 37°C, 65% humidity and 5% CO_2_. Illumination was provided by an X-Cite lamp (series 120, Lumen Dynamics Group), and images were recorded by a Coolsnap HQ camera (Photometrics). Sequential images were acquired every 5 min. Analysis was carried out using Imaris v9 (Bitplane), all cells were tracked and the averaged speed and track length analyzed. The brightfield frames are shown, with the identified tracks, color-coded based on average speed, shown below.

Given that Lphn1 is highly expressed in RG cells, whereas its binding partners Teneurins and FLRTs are present in migrating neurons (Figures 3 and S3), we addressed the complexity of Lphn1 function in the context of neuron-RG cell interactions. Cortical migration relies on a delicate balance between neuron-neuron and neuron-RG fiber interactions. Indeed, altering glial-guided neuronal migration affects neuronal dynamics and morphology as well as their tangential dispersion (Valiente et al., 2011).

We chose arrays of parallel aligned nanofibers to mimic the fibrillary environment of RG cells (Schnell et al., 2007, Vasita and Katti, 2006). As a source of neurons, we used cortical explants that, after being positioned onto nanofibers, displayed directed axon growth and cell migration along the fibers (Figures 4C and S4E). Neurons exiting the explants displayed similar features as observed *in vivo*, such as transitioning from multipolar to bipolar morphology prior to migrating along nanofibers and transient branching of their leading process, as described recently (Martínez-Martínez et al., 2019; Figure 4D; Video S2). Neurons occasionally switched between neighboring fibers (Figure 4E) but produced mostly linear paths, as observed *in vivo* (Figure 4F; Video S2). Using semi-automatic analysis (Figure S4E), we found that neurons migrated shorter distances on nanofibers coated with Lphn1 (Lec-Olf) compared with FC control protein (Figures 4G, 4H, and S4F). Non-Teneurin (TL) and non-FLRT-binding (FL) Lphn1 (Lec-Olf) mutant proteins were equally effective in slowing neuron migration. The double mutant Lphn1^TL-FL^ (Lec-Olf) resulted in reduced effects on neuron migration compared with the wild-type protein, indicating that additive or coincident binding of Teneurins and FLRTs affects neuron migration. The fact that this mutant produces partial rescue of the migration effect suggests that other interactions may also play a role; e.g., a weak glycan interaction site has been reported on mLphn1 Lec, with K_d_ values in the millimolar range (Vakonakis et al., 2008). The mean axon length and the percentage of explants producing migration were similar under all conditions (Figures 4I, 4J, S4F, and S4G). In summary, these results indicate that Lphn1 delays cortical cell migration by binding Teneurins and FLRTs while having no effect on their axon growth.

Video S2. Time-Lapse Analysis of Electroporated Cortical Neurons Migrating on Nanofibers and Time-Lapse Analysis of Cortical Neurons Migrating on Nanofibers, Related to Figure 4 First movie: Time-lapse analysis of electroporated cortical neurons migrating on nanofibers. We electroporated mouse embryos at E13.5 with pCAG-Ires-GFP and peformed explant cultures from the cortex 2 days later (E15.5). Explants were cultured on 6-well plates containing aligned nanofibers (700nm width, Sigma) coated with 40μg/ml of FC (control protein) and 100μg/ml of poly-D-lysine overnight at 37°C. The next day the plate was washed three times with PBS and coated with 20 μg/ml of laminin for ∼2hours at 37°C. Explants were cultured in neurobasal, supplemented with B27 and 0.4% methylcellulose. After 4 hours in culture, the explants were imaged with a Zeiss Axiovert 200M microscope equipped with a temperature-controlled carbon dioxide incubation chamber set to 37°C, 65% humidity and 5% CO_2_. Illumination was provided by an X-Cite lamp (series 120, Lumen Dynamics Group), and images were recorded by a Coolsnap HQ camera (Photometrics). Sequential images were acquired every 6 min. The video shows a GFP expressing neuron (in red) exiting the explant and migrating along the nanofiber. Second movie: Time-lapse analysis of cortical neurons migrating on nanofibers. We cultured cortical explants from E15.5 mouse embryos on 6-well plates containing aligned nanofibers (700nm width, Sigma) coated with 40μg/ml of FC (control protein) and 100μg/ml of poly-D-lysine overnight at 37°C. The next day the plate was washed three times with PBS and coated with 20 μg/ml of laminin for ∼2 hours at 37°C. Explants were cultured in neurobasal, supplemented with B27 and methylcellulose. After 4 hours in culture, the explants were imaged with a Zeiss Axiovert 200M microscope equipped with a temperature-controlled carbon dioxide incubation chamber set to 37°C, 65% humidity and 5% CO_2_. Illumination was provided by an X- Cite lamp (series 120, Lumen Dynamics Group), and images were recorded by a Coolsnap HQ camera (Photometrics). Sequential images were acquired every 6 min. The video shows brightfield images of neurons exiting the explant and migrating on the nanofibers. The video on the right shows the tracked path of selected neurons.

### Latrophilin Binding of Teneurins and FLRTs Is Repulsive for Embryonic Cortical Neurons

**Figure 5:**
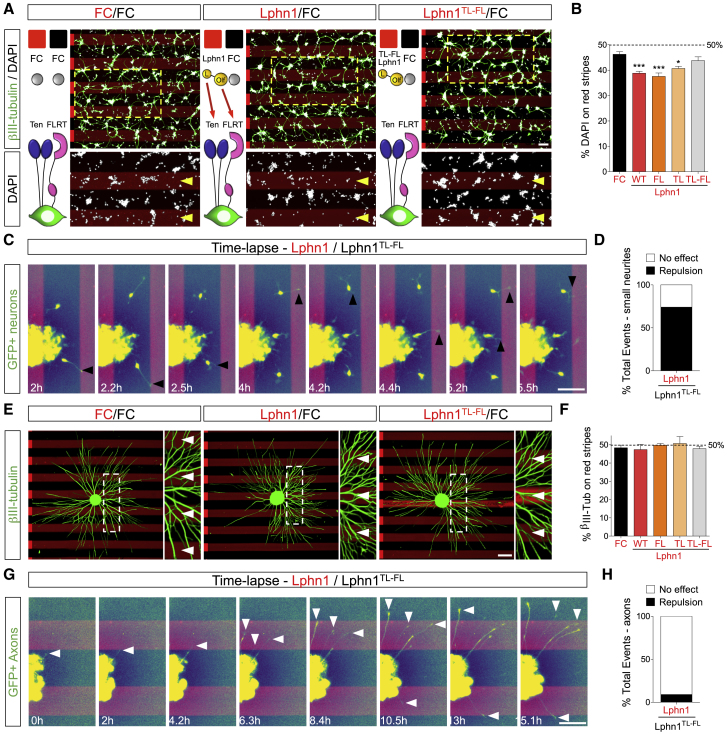
Latrophilin1 Interaction with Teneurins and FLRTs in *Trans* Induces Repulsion (A) E15.5 dissociated cortical neurons were grown on alternate stripes containing FC (black) and Lphn1 Lec-Olf proteins (red). Neurons were stained with anti- β-III-tubulin to visualize neurites (green) and nuclei (DAPI, white). In the magnified inset images, the red Lphn-containing stripes are indicated by yellow arrowheads. (B) The percentage of DAPI+ pixels on red stripes was quantified. n = 3 different experiments. ^∗^p < 0.05, ^∗∗^p < 0.01, ^∗∗∗^p < 0.001, one-way ANOVA test with Tukey’s post hoc analysis. (C) GFP+ neurons exiting cortical explants grown on alternating Lphn1 (red) and Lphn1^TL-FL^ (black) stripes. Snapshots from a time-lapse experiment are shown. Neurons prefer to migrate on Lphn1^TL-FL^ in these experiments. A repulsive event was defined as a contact between a small neurite and Lphn1 stripes lasting less than 3 frames. Black arrowheads indicate repulsive events. (D) Quantification of the data shown in (C); n > 30 contacts. (E) E15.5 cortical explants were grown on stripes as in (A) and stained with anti- β-III-tubulin to visualize axons. (F) Quantification of data shown in (E). n = 3 different experiments. (G) GFP+ axons exiting cortical explants grown on alternating Lphn1 (red) and Lphn1^TL-FL^ (black) stripes. Snapshots from a time-lapse experiment are shown. No preference for black or red stripes was observed. A repulsive event was defined as a contact between an axon and Lphn1 stripes lasting less than 3 frames. White arrowheads indicate growth cones that are not repelled from Lphn1 stripes. (H) Quantification of the data shown in (G); n > 20 contacts. Scale bars represent 300 μm (A), 200 μm (C and G), and 200 μm (E).

**Figure S5:**
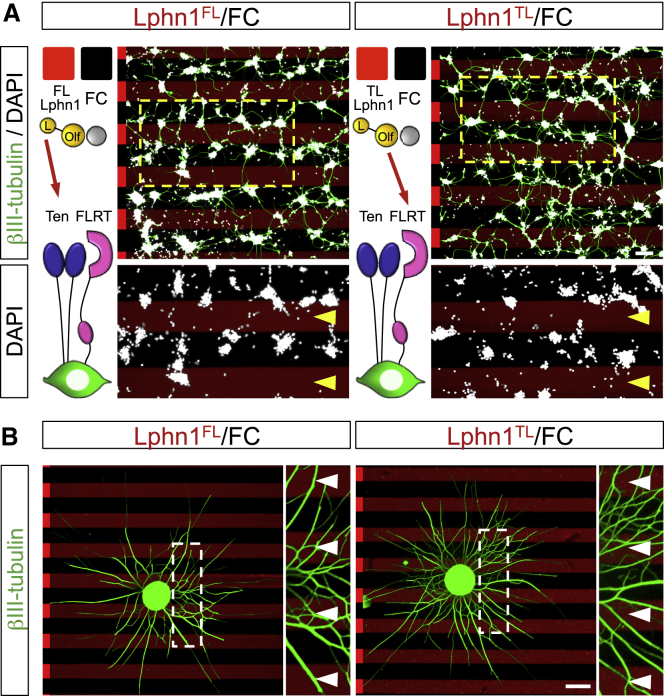
Latrophilin1 Interaction with Teneurins and FLRTs in *Trans* Induces Repulsion, Related to Figure 5 (A) E15.5 dissociated cortical neurons were grown on alternate stripes containing FC (black stripes) and wild-type or mutant TL or FL murine Lphn1 Lec-Olf protein (red stripes). Neurons were stained with anti-beta-III-tubulin to visualize neurites (green) and DAPI (white). High magnification showing the location of nuclei (DAPI, white) on stripes is shown at the bottom. Red stripes, which contain Lphn protein, are indicated with yellow arrowheads. After imaging, the percentage of DAPI+ pixels on red stripes was quantified and it is shown in Figure 5B. (B) E15.5 cortical explants were grown on the same stripes as in (A) and the quantification of the results is shown Figure 5F. White arrow heads indicate the red stripes which contain Lphn1 mutant protein. Scale bars represent 20 μm (A) and 200 μm (B).

The mechanism by which cell surface proteins decrease cell migration speed could be due to a change in the balance of adhesion versus repulsion. Modulating either can lead to a reduction of migration in other systems. For example, during *Xenopus* gastrulation, migrating cells alternate between attachment and detachment. Reducing contact repulsion, in that case mediated by ephrinB-EphB signaling, increases attachment and reduces cell motility (Rohani et al., 2011). Conversely, increasing ephrinB-EphB repulsion induces cell detachment (Wen and Winklbauer, 2017), also affecting migration. Using stripe assays, we had shown previously that Lphn3 (Lec-Olf) repelled embryonic cortical neurons (Jackson et al., 2015). Here we found that Lphn1 (Lec-Olf) is also mildly repulsive for cortical neurons (Figures 5A, 5B, and S5A). Similar to the nanofiber assay described above, using stripe assays, we find that non-Teneurin (TL) and non-FLRT-binding (FL) Lphn1 single mutants are also repulsive. The Lphn1^TL-FL^ double mutant produces no response compared with the control. Time-lapse imaging of dissociated cortical neurons and GFP electroporated neurons choosing between alternate stripes of Lphn1 (Lec-Olf) and the TL-FL mutant showed that cell bodies and small neurites prefer the mutant (Figures 5C and 5D; Videos S3 and S4). These data suggest that the Lphn1-induced repulsion from stripes is due to its interaction with Teneurins and FLRTs in *trans*. Lphn1 was not repulsive for cortical axons (Figures 5E, 5F, and S5B), which is also in agreement with the nanofiber assay. Also, GFP electroporated axons were not repelled by Lphn1 in time-lapse experiments. The growth cones of these axons did not show a preference for the Lphn1 (Lec-Olf) wild type or the TL-FL mutant (Figures 5G and 5H; Video S4). In summary, these results suggest that Lphn1 is repulsive for migrating cortical cells through an interaction with Teneurins and FLRTs but not for their axons.

Video S3. Time-Lapse Analysis of Dissociated Cortical Neurons on Lphn1 and Lphn1^TL-FL^ Stripes, Related to Figure 5 Upper left movie: Dissociated cortical neurons (E15.5) were plated on stripes. 50μg/ml of Lphn1 protein was mixed with Alexa594-conjugated anti-hFC antibody (Invitrogen) in PBS. Proteins were injected into matrices (90 μm width) and placed on 60 mm dishes (Knöll et al., 2007), resulting in red-fluorescent stripes. After 30 min incubation at 37°C, the dishes were washed with PBS and the matrices removed. The dishes were coated with 50 μg/ml of Lphn1^TL-FL^ protein mixed with anti-hFC for 30 min at 37°C, washed three times with PBS, and coated with 20 μg/ml laminin for ∼2 hours at 37°C. After 2 hours in culture, neurons were imaged with a Zeiss Axiovert 200M microscope equipped with a temperature-controlled carbon dioxide incubation chamber set to 37°C, 65% humidity and 5% CO_2_. Illumination was provided by an X-Cite lamp (series 120, Lumen Dynamics Group), and images were recorded by a Coolsnap HQ camera (Photometrics). Sequential images were acquired every 7 min. The video shows dissociated cortical neurons forming clusters over time and being repelled from Lphn1-containing red stripes. The bottom left movie shows the tracks of individual cells (small gray circles) and also the formation of cell clusters (indicated by bigger circles), using analysis with Imaris v9 (Bitplane). The upper right movie shows the tracked cells and clusters on the stripes that were used in the quantification shown in the bottom right movie. The percentage of tracked neurons on Lphn1 (red) stripes shows that there is a reduction of neurons on these stripes over time.

Video S4. Time-Lapse Analysis of GFP+ Cortical Neurons on Lphn1 and Lphn1^TL-FL^ Stripes and Time-Lapse Analysis of GFP+ Cortical Axons on Lphn1 and Lphn1^TL-FL^ Stripes, Related to Figure 5 First movie: Time-lapse analysis of GFP+ cortical neurons on Lphn1 and Lphn1^TL-FL^ stripes. We electroporated mouse embryos at E13.5 with pCAG-IRES-GFP and peformed cortical cultures from the cortex 2 days later (E15.5). Cortical explants were plated on stripes. To generate the stripe pattern, 50μg/ml of Lphn1 protein was mixed with Alexa594-conjugated anti-hFC antibody (Invitrogen) in PBS. Proteins were injected into matrices (90 μm width) and placed on 60 mm dishes (Knöll et al., 2007), resulting in red-fluorescent stripes. After 30 min incubation at 37°C, the dishes were washed with PBS and the matrices were removed. The dishes were coated with 50 μg/ml of Lphn1^TL-FL^ protein mixed with anti-hFC for 30 min at 37°C, washed three times with PBS, and coated with 20 μg/ml laminin for ∼2 hours at 37°C. After 2 hours in culture, the neurons were imaged with a Zeiss Axiovert 200M microscope equipped with a temperature-controlled carbon dioxide incubation chamber set to 37°C, 65% humidity and 5% CO_2_. Illumination was provided by an X-Cite lamp (series 120, Lumen Dynamics Group), and images were recorded by a Coolsnap HQ camera (Photometrics). Sequential images were acquired every 10 min. The video shows GFP+ cortical neurons exiting the explant. The majority of small neurites from these neurons preferred to grow on the Lphn1^TL-FL^ stripes. Second Movie: Time-lapse analysis of GFP+ cortical axons on Lphn1 and Lphn1^TL-FL^ stripes. We electroporated mouse embryos at E13.5 with pCAG-IRES-GFP and peformed cortical cultures from the cortex 2 days later (E15.5). Cortical explants were plated on stripes. To generate the stripe pattern, 50μg/ml of Lphn1 protein was mixed with Alexa594-conjugated anti-hFC antibody (Invitrogen) in PBS. Proteins were injected into matrices (90 μm width) placed on 60 mm dishes (Knöll et al., 2007), resulting in red-fluorescent stripes. After 30 min incubation at 37°C, dishes were washed with PBS and matrices were removed. The dishes were coated with 50 μg/ml of Lphn1^TL-FL^ protein mixed with anti-hFC for 30 min at 37°C, washed three times with PBS, and coated with 20 μg/ml laminin for ∼2 h at 37°C. After 2 hours in culture, the neurons were imaged with a Zeiss Axiovert 200M microscope equipped with a temperature-controlled carbon dioxide incubation chamber set to 37°C, 65% humidity and 5% CO_2_. Illumination was provided by an X-Cite lamp (series 120, Lumen Dynamics Group), and images were recorded by a Coolsnap HQ camera (Photometrics). Sequential images were acquired every 10 min. The video shows GFP+ cortical axons exiting the explant. They display normal growth and extension when contacting Lphn1 stripes. In the same video, a cortical neuron exiting the explant is repelled when its small neurites interact with Lphn1 stripes.

### Teneurins and Latrophilins Control Radial Migration *In Vivo*

**Figure 6:**
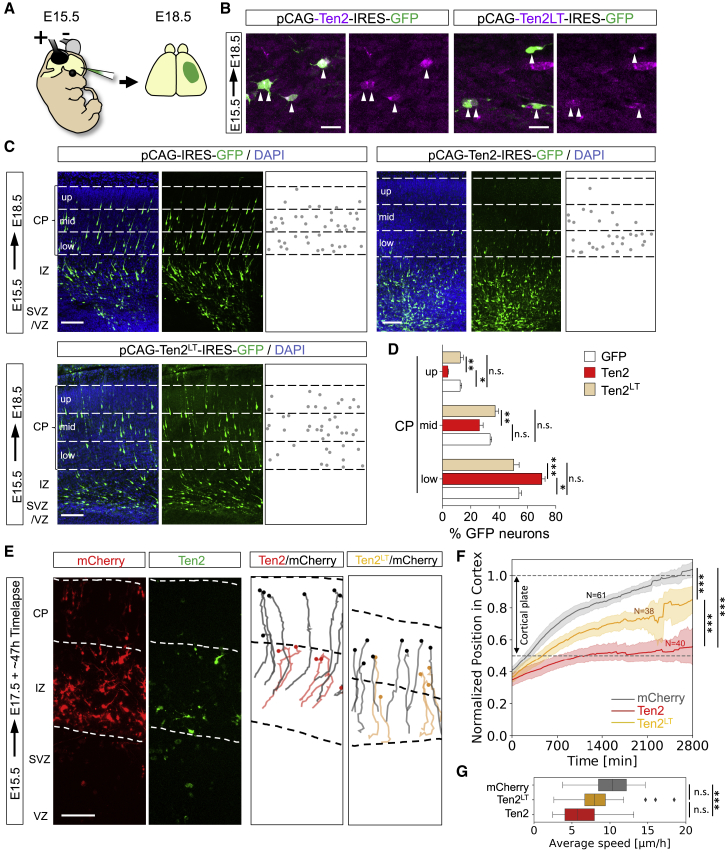
Latrophilin Interaction with Teneurins Delays Neuron Migration (A) Schematic of *in utero* electroporation (IUE) performed at E15.5. (B) IUE of pCAG-Ten2-IRES-GFP or pCAG-Ten2^LT^-IRES-GFP was performed and analyzed at E18.5. Hemagglutinin (HA)-tagged Ten2 and Ten2^LT^ protein expression in neurons was confirmed by immunostaining with anti-HA (magenta). Ten2 and Ten2^LT^ expression coincides with expression of the reporter GFP (green). (C) Coronal sections after IUE. The CP was subdivided into 3 bins (up, mid, and low), and the number of GFP+ neurons in each bin was quantified. (D) Quantification of the data shown in (C). n = 6 GFP, n = 7 Ten2, and n = 10 Ten2^LT^ electroporated brains. ^∗^p < 0.05, ^∗∗^p < 0.01, ^∗∗∗^p < 0.001, one-way ANOVA test with Tukey’s post hoc analysis. (E) For time-lapse imaging of live brain slices, sections were performed at E17.5, 2 days after IUE. Neurons were tracked as they migrated from the IZ to the CP (representative tracks are shown on the right). Dashed lines indicate the positions of the IZ and CP. (F) Quantification of the data shown in (E). n = 61 mCherry-expressing, n = 38 Ten2^LT^-expressing, and n = 40 Ten2-expressing neurons from 3 experiments. ^∗∗∗^p < 0.001, one-way ANOVA test with Tukey’s post hoc analysis. (G) The average speed of the tracked neurons is shown as a whisker plot. Ten2- but not Ten2^LT^-overexpressing neurons migrate significantly slower compared with control neurons. ^∗∗∗^p < 0.001, one-way ANOVA test with Tukey’s post hoc analysis. Scale bars represent 20 μm (B), 100 μm (C), and 80 μm (E).

**Figure S6:**
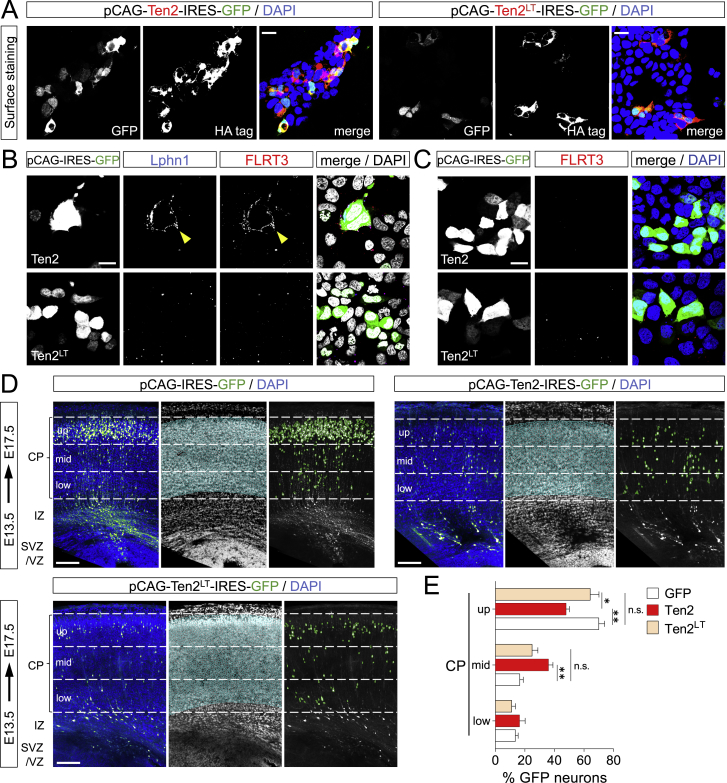
Latrophilin Interaction with Teneurins Delays Neuron Migration, Related to Figure 6 (A) HA-tag surface staining was performed on HEK293 cells expressing the plasmids used for in-utero-electroporation (IUE) assays, pCAG-Ten2-IRES-GFP and pCAG-Ten2^LT^-IRES-GFP. Ten2 and Ten2^LT^ HA-staining coincides with GFP-expressing cells (green), validating correct cell surface expression. (B) HEK293 cells expressing pCAG-Ten2-IRES-GFP and pCAG-Ten2^LT^-IRES-GFP where incubated with immuno-clustered Lphn1 protein (blue) and immuno-clustered FLRT3 protein (red) for 20min at room temperature. Fixed cells were imaged. Ten2, but not the LT mutant, binds to Lphn1 which in turn binds FLRT3 (yellow arrowhead). (C) HEK293 cells expressing pCAG-Ten2-IRES-GFP and pCAG-Ten2^LT^-IRES-GFP where incubated with immuno-clustered FLRT3 protein (red). Ten2 and its mutant Ten2^LT^ do not bind FLRT3 in the absence of Latrophilin. (D) Coronal sections of E17.5 cortex after IUE at E13.5 with pCAG-IRES-GFP, pCAG-Ten2-IRES-GFP or pCAG-Ten2^LT^-IRES-GFP. The cortical plate (CP) is defined based on the DAPI staining (nuclei outlined in cyan) and subdivided in 3 bins (up, mid and low). GFP^+^ neurons localized within the CP were automatically identified (outlined in green) and the percentage in each bin was quantified. (E) Quantification of data shown in (D). n = 7 GFP, n = 7 Ten2, and n = 8 Ten2^LT^ electroporated brains. ^∗^p < 0.05, ^∗∗^p < 0.01, one-way ANOVA test with Tukey’s post hoc analysis. Scale bars represent 20 μm (A-C) and 150 μm (D).

**Figure 7:**
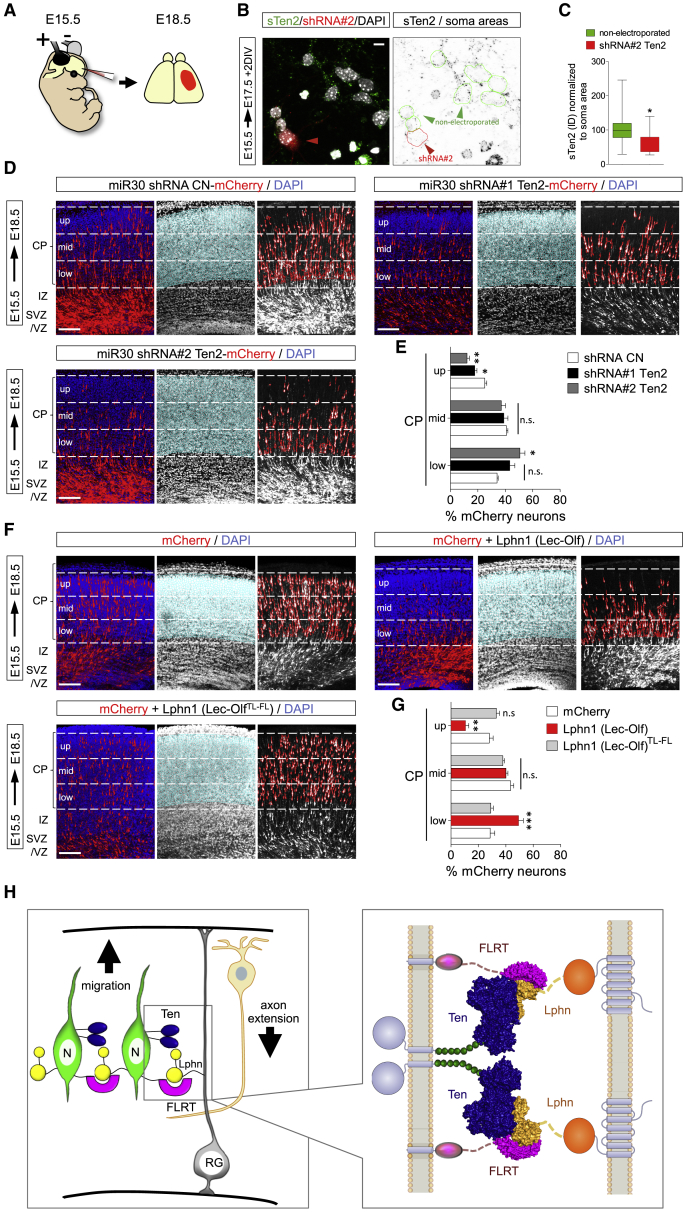
Loss of Teneurin Delays Neuronal Migration (A) Schematic of IUE performed at E15.5. (B) Neurons were electroporated with pCAG-mCherry and pCAG-miR30 containing shRNA#2 for murine Ten2 (red), harvested and plated as dissociated cultures at E17.5, cultured for 2 DIV, and immunostained for surface Ten2 (green) (left image). A magnified view shows cell nuclei (DAPI) of non-electroporated neurons (green lines) and mCherry+ shRNA#2+ neurons (red lines) (right image). (C) Quantification of the data shown in (B). n > 40 non-electroporated and n > 13 shRNA#2. ^∗^p < 0.05, one-way ANOVA test with Tukey’s post hoc analysis. (D) Coronal sections of an E18.5 cortex previously electroporated with pCAG-mCherry and a pCAG-miR30 vector coding for shRNA control (CN), shRNA#1, or shRNA#2. The latter two constructs target murine Ten2. The cortical plate was subdivided into 3 bins (up, mid, and low), and the number of mCherry+ neurons in each bin was quantified. (E) Quantification of the data shown in (D). n = 5 CN, n = 6 shRNA#1, and n = 5 shRNA#2 electroporated brains. ^∗^p < 0.05, ^∗∗^p < 0.01, one-way ANOVA test with Tukey’s post hoc analysis. (F) Coronal sections of an E18.5 cortex electroporated to express mCherry alone or together with the secreted version of wild-type or TL-FL mutant Lphn1 (Lec-Olf). The cortical plate was subdivided into 3 bins (up, mid, and low), and the number of mCherry+ neurons in each bin was quantified. (G) Quantification of the data shown in (F). n = 7 mCherry, n = 6 Lphn1 (Lec-Olf), and n = 6 Lphn1 (Lec-Olf)^TL-FL^ electroporated brains. ^∗^p < 0.05, ^∗∗^p < 0.01, one-way ANOVA test with Tukey’s post hoc analysis. (H) Schematic depicting a model of Teneurin, FLRT, and Latrophilin in radial cortical migration. Scale bars represent 15 μm (B) and 100 μm (D).

**Figure S7:**
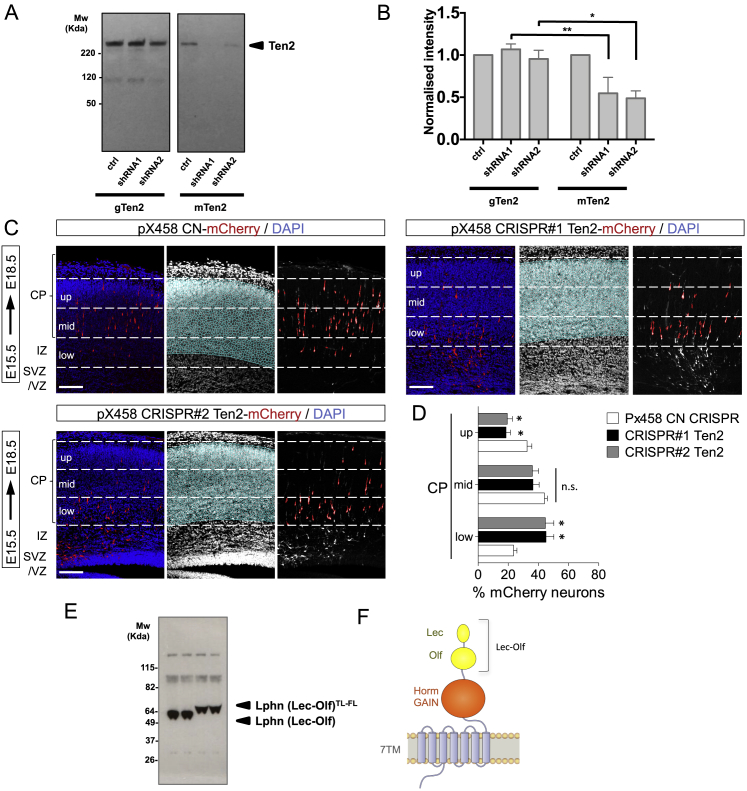
Loss of Teneurin Delays Neuronal Migration, Related to Figure 7 A) HEK293T cells were co-transfected with pCAG-miR30 containing shRNA or control inserts, and either chicken Ten2 (gTen2), or murine Ten2 (mTen2). The sequences chosen were selected to match the murine gene only, not chicken *ten2*. Effective knock-down was observed only for overexpressed murine Ten2. (B) Quantification of data shown in (A). The expression was quantified using ImageJ and the values for gTen2 and mTen2 shRNA-co-transfected samples were normalized using the control gTen2 or mTen2 intensities, respectively. ^∗^p = 0.0168, ^∗∗^p = 0.0054, one-way ANOVA test with Tukey’s post hoc analysis. (C) Coronal sections of E18.5 cortex after IUE at E15.5 with CRISPR control, Ten2 CRISPR#1 and CRISPR#2 using the pX458 plasmid with pCAG-mCherry. The cortical plate (CP) is subdivided into 3 bins (up, mid and low) and the number of mCherry+ neurons in each bin was quantified. (D) Quantification of data shown in (C). n = 6 CN, n = 5 CRISPR#1, and n = 4 CRISPR#2 electroporated brains. ^∗^p < 0.05, one-way ANOVA test with Tukey’s post hoc analysis. (E) Western blot to validate the expression of *pCAGIG* Myc-tagged Lphn1 (Lec-Olf) wild-type and TL-FL mutant, used in IUE experiments (Figures 7F and 7G). The mutant contains two additional N-linked glycosylation sites, which block FLRT and Teneurin binding. It therefore runs slightly higher on the gel compared to the wild-type. (F) Schematic showing the portion of Lphn1 that is included in the Lec-Olf construct (see also Figure 2B). Scale bars represent 150 μm (C).

Having established that Lphn1 binding of Teneurins and FLRTs is repulsive for migrating neurons *in vitro*, we next addressed their function in the developing cortex. Using *in utero* electroporation (IUE) at E15.5, we overexpressed full-length Ten2 plus GFP, the non-Latrophilin-binding Ten2^LT^ plus GFP, or GFP control in cortical cells and analyzed their distribution at E18.5 (Figure 6A). We confirmed overexpression of Ten2 and Ten2^LT^ in migrating neurons (Figure 6B) and their correct localization and ligand-binding abilities at the cell surface (Figures S6A–S6C). We found that Ten2 overexpression in cortical migrating neurons delayed their migration. This delay was not observed when overexpressing Ten2^LT^ (Figures 6C and 6D), suggesting a functional role of Teneurin-Latrophilin interaction. Similar results were obtained when targeting cortical neurons at earlier stages (E13.5) (Figures S6D and S6E), which is consistent with the early expression of these proteins (Figures S3A–S3C). We performed live imaging of embryonic cortex *ex vivo* to find out whether Ten2 overexpression affects cortical migration. For these experiments, brains were sliced and imaged 48 h after electroporation with Ten2/GFP or Ten2^LT^/GFP and mCherry to also label control neurons. Tracking of migrating neurons revealed that Ten2-expressing neurons migrated slower and remained longer in the IZ compared with control neurons (Figures 6E–6G; Video S5). The effects on neuronal migration were more modest when overexpressing Ten2^LT^ mutant protein (Figures 6E–6G; Video S5). In a separate set of experiments, we knocked down endogenous Ten2 in E15.5 cortices by embedding small hairpin RNA (shRNA) target sequences into the pCAG-miR30 vector system (Matsuda and Cepko, 2007; Figures 7A–7C, S7A, and S7B). Analysis at E18.5 showed reduced migration of these neurons (Figures 7D and 7E). Similar results were obtained when Ten2 expression was targeted by CRISPR using the pX458 system (Ran et al., 2013; Figures S7C and S7D). We also expressed a secreted version of Lphn1 (Lec-Olf) to compete with its endogenous ligands at E15.5, and this produced a strong delay in neuronal migration. This effect was not observed when using the mutant (TL-FL) construct (Figures 7F, 7G, S7E, and S7F). These results show that Latrophilin interactions are essential for cortical neuron migration *in vivo* (Figure 7H).

Video S5. Time-Lapse Analysis of mCherry (Control)- and Ten2-Overexpressing Neurons and Time-Lapse Analysis of mCherry (Control)- and Ten2^LT^-Overexpressing Neurons, Related to Figure 6 First Movie: Time-lapse analysis of mCherry (control) and Ten2 overexpressing neurons. Live imaging of cortical neurons electroporated with pCAG-mCherry and pCAG-Ten2-IRES-GFP at E15.5, and cultured at E17.5. Individual tracks for mCherry (red) or Ten2 (green) expressing neurons are shown. Ten2 overexpression in cortical neurons delayed their migration. Acquisition interval, 20 min. Scale bar, 100μm. Second Movie: Time-lapse analysis of mCherry (control) and Ten2^LT^ overexpressing neurons. Live imaging of cortical neurons electroporated with pCAG-mCherry and pCAG-Ten2^LT^-IRES-GFP neurons at E15.5, and cultured at E17.5. Individual tracks for mCherry (red) or Ten2^LT^ (green) expressing neurons are shown. Acquisition interval, 20 min. Scale bar, 100μm.

## Discussion

Teneurins are highly conserved, with chicken and murine Teneurin 2 sharing 93% and 99% sequence identity with human Teneurin 2, respectively. The structural mechanism of how Teneurin binds to Latrophilins has long remained enigmatic, and previously available knowledge was lacking detailed structural information. The data presented here demonstrate that the Latrophilin-binding site on Teneurin comprises separate adjacent binding motifs located across the tiers of the spiraling β-sheet of the YD shell. Superposition with the FLRT2 LRR, as found previously bound to Lphn3, gives a compact ternary model that still involves the Teneurin YD shell domain rather than other Teneurin domains. The interface between the two bottom tiers of the Teneurin YD shell is of structural interest because it enables passage of the internal linker through the YD shell and into the ABD. In the related bacterial TcB/TcC toxins (Busby et al., 2013, Meusch et al., 2014), the equivalent interface area between the tiers of the YD shell is formed by two separate subunits of the toxin protein (Jackson et al., 2018), suggesting that they come together post-translationally in bacteria. Given its geometry, this area of Teneurin must undergo significant conformational changes during protein folding and, therefore, may be inherently mobile. Latrophilin binds across these tiers of the Teneurin YD shell like a clamp and appears to stabilize the fold of this structural module.

Previous studies have emphasized that the inclusion of an alternatively spliced loop in the Teneurin NHL loop regulates Teneurin interactions, with some reports suggesting that inclusion of the loop inhibits Latrophilin binding (Li et al., 2018, Sando et al., 2019). Others found that the presence of this loop does not affect Teneurin-Latrophilin binding but that it promotes homophilic interactions of Teneurin together with an alternatively spliced sequence in the Teneurin EGF domains (Berns et al., 2018). Our Teneurin constructs include the alternatively spliced loop in the NHL domain but not that in the EGF domains. In agreement with (Berns et al., 2018), the constructs do not promote homophilic interaction, and they bind Latrophilins. The crystallographic data explain these binding results; the NHL domain does not engage with Latrophilin directly. Using the new structural data, we revealed a molecular map of how Teneurin, Latrophilin, and FLRT termini interact *in vitro* and designed a toolkit of mutants that control the individual interactions formed by these receptors. These mutant tools are used to interrogate the functions of specific interactions in cellular and *in vivo* settings.

Teneurins have recently emerged as key regulators of synaptic wiring (Berns et al., 2018, Dharmaratne et al., 2012, Glendining et al., 2017, Hong et al., 2012, Mosca and Luo, 2014, Mosca et al., 2012, Silva et al., 2011, Vysokov et al., 2018, Young et al., 2013). This highly evolved function contrasts with the evolutionarily early origin of Teneurins in unicellular organisms that lack a nervous system (Jackson et al., 2018, Tucker et al., 2012). In these organisms, Teneurin-like proteins likely engage in other types of cell-cell or cell-extracellular matrix interactions. This is consistent with studies in worms, where Teneurin (Ten-1) is essential for gonad epithelialization and basement membrane integrity (Trzebiatowska et al., 2008).

Here we show that, in the mammalian nervous system, Teneurins play important roles at earlier time points, much before synapse development. They direct the migration of embryonic cortical neurons, possibly by regulating their interaction with the radial glia scaffold. Although Teneurins and FLRTs are mainly expressed in migrating neurons, Latrophilins are expressed in neurons and RG cells. Mechanistically, the migrating neuron co-expresses Teneurins and FLRTs, and these two proteins could therefore bind Latrophilins coincidently in *trans* on opposing RG cells or other neurons (Figure 7H). This configuration is similar to the one proposed for their synaptogenic function: Teneurins and FLRTs on the pre-synaptic side interact with Latrophilins in *trans* on the post-synaptic side (Sando et al., 2019). Other configurations are conceivable, and it is possible that different context-dependent complexes form transiently as the cells migrate through their complex environment. Cell type-specific manipulation of expression of Teneurins, FLRTs, and Latrophilins will be needed to unravel the most important configurations for cortical neuron migration *in vivo*. Given that the formation of synapses is an adhesive/attractive process, we were surprised to find that the interaction between Teneurins, FLRTs, and Latrophilins during cell migration is repulsive. The delay in cell migration induced by overexpression of Teneurin could be caused by loss of traction rather than an increase in adhesion. Such a dual role in repulsive cell guidance and synaptogenesis is not without precedent. Erythropoietin-producing human hepatocellular (Eph) receptor/ephrin signaling has long been reported to mediate these processes (Henderson and Dalva, 2018, Kania and Klein, 2016), but despite intense research, the underlying signaling mechanisms that convert repulsion into synapse formation are poorly understood. Previous studies have identified different molecules promoting adhesion of migrating neurons to RG fibers such as connexin26/43 (Elias et al., 2007), focal adhesion kinase (FAK) (Valiente et al., 2011), and N-cadherin (Shikanai et al., 2011). However, the molecules that mediate repulsion between neurons and RG fibers remain largely unknown.

Given that the synaptogenic functions of Teneurins and FLRTs require their localization on the pre-synaptic side, we were surprised to see that cortical axons showed no response toward Latrophilins. The easiest explanation would have been that, during the migratory phase, Teneurins and FLRTs are excluded from the axonal compartment and that these proteins are induced in axon terminals during synaptogenesis. However, we find that Ten2 and FLRT3 are co-expressed and show uniform distribution on the cell surface during the migration phase. These results suggest that the differential responses—i.e., repulsion of somata and small neurites and adhesion of axon terminals—are the result of differences in the downstream signaling pathways between the somatodendritic and axonal compartments. Similar results have been shown for Semaphorin 3A, which is attractive for cortical dendrites but repulsive for axons because of asymmetrically localized guanylate cyclase (Polleux et al., 2000). The highly polarized structure of migrating neurons could also contribute to the different response between its dendrites and axons. The leading process of migrating neurons seems to preferentially mediate the interactions toward RG fibers (Elias et al., 2007). These increased contacts induce polarization of downstream signaling molecules, such as RhoA, being recruited to the leading process and Rac1 to the opposed neurite that will become the axon (Xu et al., 2015).

*In vivo*, overexpression and knockdown of Ten2 in cortical neurons delayed their migration toward the CP, and this effect was not observed when overexpressing the Ten2 mutant defective in Lphn binding. The impairing effect of tampering with the Ten2 levels on migrating neurons is reminiscent of other receptors regulating cell migration, such as Neuroligin 2 (Heng et al., 2008). FLRT loss-of-function and gain-of-function experiments also resulted in abnormal migration of cortical neurons (Seiradake et al., 2014). The need for finely balanced levels of Ten2 in migrating neurons is a likely reason why attempts at loss-of-function rescue experiments, in which we tried to simultaneously knock down endogenous Ten2 and overexpress a resistant version by IUE, failed. It is likely that the levels of resistant Ten2 overexpressed in these neurons is high, more similar to our overexpression experiments than the correct endogenous levels. We complement our loss-of-function results with a separate *in vivo* assay where we overexpressed the secreted portion of the Latrophilin ectodomain that interacts with FLRTs and Teneurins (Lec-Olf) to effectively compete with the interactions of endogenous Lphn with its binding partners and delay cell migration. Regarding the downstream signaling pathways that mediate repulsion, very little is known. We have shown previously that Lphn-FLRT and FLRT-Unc5 interactions can trigger repulsive responses in cortical neurons *in vitro* (Jackson et al., 2015, Yamagishi et al., 2011). In future work, it would be interesting to assess the possible role of Unc5 receptors in the context of a Teneurin-FLRT-Latrophilin complex. Unc5 are classic repulsive cell guidance receptors and form supercomplexes with Latrophilins and FLRTs (Jackson et al., 2016). Recruitment of Unc5 to a Teneurin-FLRT-Latrophilin complex may enhance repulsive signaling.

In conclusion, our crystal structures revealed the main binding site for Latrophilin on the Teneurin YD shell domain. Latrophilin-Teneurin and Latrophilin-FLRT interactions give rise to a ternary complex and can be controlled by specific mutations in the Lec and Olf domains, respectively. Latrophilin binding of Teneurins and FLRTs, previously shown to engage neurons in synapse formation, slows down the migration of embryonic cortical neurons by contact repulsion. In our paradigm, the repulsive response involves the somatodendritic compartment of neurons without affecting their axon growth and extension, which are initiated concomitantly with migration. Teneurins and Latrophilins show broad expression during brain development, and so the reported mechanism could also be involved in controlling neuronal migration in other brain regions.

## STAR★Methods

### Key Resources Table

**Table:** 

REAGENT or RESOURCE	SOURCE	IDENTIFIER
**Antibodies**		

Anti-HA	SIGMA-Aldrich	Cat#H3663; RRID: AB_262051
Anti-FLAG	SIGMA-Aldrich	Cat#F1804; RRID: AB_262044
Anti-Myc	Abcam	Cat#ab19233; RRID: AB_731656
Cy3-labeled anti-mouse	Abcam	Cat#ab97035; RRID: AB_10680176
Anti-chicken A568	Abcam	Cat#ab175711
Anti-6xHis Tag	Life Technologies	Cat#372900; RRID: AB_2533309
Anti-6xHis-tag FITC	Abcam	Cat#ab3554; RRID: AB_303901
Anti-6xHist-tag DyLight 650	Abcam	Cat#ab117504; RRID: AB_11001222
Ani-FLAG	SIGMA-Aldrich	Cat#F9291; RRID: AB_439698
Cy3-conjugated αFc	Life Technologies	Cat#A11014
Anti-human IgG	Jackson ImmunoResearch	Cat#109-005-098; RRID: AB_2337541
Rabbit monoclonal anti-beta-III tubulin antibody	SIGMA-Aldrich	Cat#ZRB1140
Cy2 anti-rabbit IgG secondary antibody	Jackson ImmunoResearch	Cat#111-225-144; RRID: AB_2338021
Anti-Teneurin-2 antibody	Novus Biological	Cat#NBP2-55763
Anti-FLRT3 antibody	R&D	Cat#AF2795; RRID: AB_2106855
Anti-GFP antibody	Life Technologies	Cat#A11122; RRID: AB_221569
Anti-His Alexa 488	Thermofisher	Cat#MA1-21315-A488; RRID: AB_2610645
Anti-goat Alexa 594	Jackson ImmunoResearch	Cat#705-585-003; RRID: AB_2340432
Anti-N-terminal Teneurin 2 antibody	R&D	Cat#AF4578; RRID: AB_10719438
Anti-goat Alexa 647	Jackson ImmunoResearch	Cat#713-606-147; RRID: AB_2340752
Mouse anti-His antibody	QIAGEN	Cat#34660; RRID: AB_2619735
Mouse anti-Pvim antibody	Abcam	Cat#ab20346; RRID: AB_445527
Rat anti-Ctip2 antibody	Abcam	Cat#ab123449; RRID: AB_10973033

**Chemicals, Peptides, and Recombinant Proteins**		

DMEM medium	Life Technologies	Cat#21969035
IMDM medium	Life Technologies	Cat#12440061
PBS (used in purification buffers in this study)	SIGMA-Aldrich	Cat#P4417
PBS (used for cell culture in this study)	Lonza	Cat#LZBE17-516F
10% FBS	Life Technologies	Cat#10270-106
1% NEAA	Life Technologies	Cat#11140035
1% L-Glutamine	Life Technologies	Cat#25030-024
Polyethylenimine	SIGMA-Aldrich	Cat#208727
0.1% Triton	CarlRoth	Cat#3051
Bovine Serum Albumin	SIGMA-Aldrich	Cat#A7906-100G
Lipofectamine LTX with PLUS reagent	Life Technologies	Cat#15338100
Neurobasal medium supplemented with B27	Invitrogen	Cat#A3582901
Methyl-cellulose	SIGMA-Aldrich	Cat#M7027
Fast green FCF stain	SIGMA-Aldrich	Cat#2353-45-9
Sepharose beads	Amersham CL-4B	Cat#17-01780-01
Protein G Fast Flow Sepharose	SIGMA-Aldrich	Cat#P3296
Low melting agarose	Biozym	Cat#840101
Penicillin Streptomycin	GIBCO	Cat#155140148
B27 Supplement	GIBCO	Cat#17504044
N-2 Supplement	GIBCO	Cat#17502001
Immu-mount	Thermofisher	Cat#10622689
Dako Mounting medium	Agilent	Cat#S3023
RNAscope Universal Pretreatment Kit	Advanced Cell Diagnostics	Cat#322380
RNAscope Fluorescent Multiplex Reagent Kit	Advanced Cell Diagnostics	Cat#320850

**Critical Commercial Assays**		

Bio-Rad protein assay	Biorad	Cat#5000001

**Deposited Data**		

Protein Data Bank	This study	PDB: 6SKE
Protein Data Bank	This study	PDB: 6SKA

**Experimental Models: Cell Lines**		

HEK293T Cells	ATCC	CRL-3216; RRID: CVCL_0063
HEK293S Cells	ATCC	CRL-3022; RRID: CVCL_A785
K-562 Cells	ATCC	CCL-243; RRID: CVCL_0004

**Experimental Models: Organisms/Strains**		

Primary cortical neurons and explants from mouse	The Jackson Laboratory (maintained at the Max-Planck Institute of Neurobiology)	C75BL/6 background

**Oligonucleotides**		

Primers are listed in the respective Method Details section within STAR Methods.		N/A
ISH: Mm-Lphn1-C1	Rnascope	Cat#319331
ISH: Mm-Lphn2-C2	Rnascope	Cat#319341
ISH: Mm-Lphn3-C3	Rnascope	Cat#317481
ISH: Mm-Tenm1-C3	Rnascope	Cat#500641
ISH: Mm-Tenm2-C2	Rnascope	Cat#552671
ISH: Mm-Tenm3-C1	Rnascope	Cat#411951
ISH: Mm-Tenm4-C1	Rnascope	Cat#555491

**Recombinant DNA**		

Plasmid: pHL-sec	Aricescu et al., 2006	Addgene plasmid #99845; RRID: Addgene_99845
Plasmid: pCAGIG	Matsuda and Cepko, 2004	Addgene plasmid #11159; RRID:Addgene_11159
Plasmid: pCAG-mir30	Matsuda and Cepko, 2007	Addgene plasmid #14758; RRID:Addgene_14758
Plasmid: PX458	Ran et al., 2013	Addgene plasmid #48138; RRID:Addgene_48138

**Software and Algorithms**		

xia2	Winter et al., 2013	http://scripts.iucr.org/cgi-bin/paper?S0907444913015308
phaser	McCoy et al., 2007	http://scripts.iucr.org/cgi-bin/paper?S0021889807021206
SWISS-MODEL	Bordoli et al., 2009	http://www.nature.com/articles/nprot.2008.197
ccp4	Winn et al., 2011	http://scripts.iucr.org/cgi-bin/paper?S0907444910045749
buster	Smart et al., 2012	http://scripts.iucr.org/cgi-bin/paper?S0907444911056058
coot	Emsley and Cowtan, 2004	http://scripts.iucr.org/cgi-bin/paper?S0907444904019158
molprobity	Davis et al., 2007	https://academic.oup.com/nar/article-lookup/doi/10.1093/nar/gkm216
Consurf	Glaser et al., 2003	https://academic.oup.com/bioinformatics/article-lookup/doi/10.1093/bioinformatics/19.1.163
Clustal Omega	Madeira et al., 2019	https://www.ebi.ac.uk/Tools/msa/clustalo/
GROMACS 2018	Abraham et al., 2015	http://www.gromacs.org/
AMBER 99SB force field	DePaul et al., 2010, Lindorff-Larsen et al., 2010, Sorin and Pande, 2005	https://academic.oup.com/nar/article-lookup/doi/10.1093/nar/gkq134
		http://wiley.com/10.1002/prot.22711
		https://linkinghub.elsevier.com/retrieve/pii/S000634950573304X
SPC/E water model	Berendsen et al., 1987	https://pubs.acs.org/doi/abs/10.1021/j100308a038
Velocity-rescaling thermostat	Bussi et al., 2007	http://aip.scitation.org/doi/10.1063/1.2408420
Parrinello–Rahman barostat	Parrinello and Rahman, 1981	http://aip.scitation.org/doi/10.1063/1.328693
Particle-Mesh Ewald method	Darden et al., 1993, Essmann et al., 1995	http://aip.scitation.org/doi/10.1063/1.464397
		http://aip.scitation.org/doi/10.1063/1.470117
LINCS algorithm	Hess, 2007	https://pubs.acs.org/doi/10.1021/ct700200b
MDAnanlysis library	Michaud-Agrawal et al., 2011	http://wiley.com/10.1002/jcc.21787
Jupyter notebook and tcl script	This paper	https://github.com/MChavent/Hbond-analysis
ImageJ (version 1.51p)	Schneider et al., 2012	https://imagej.net/Welcome
SMLM image reconstruction	Grull et al., 2011	http://www.kip.uni-heidelberg.de/user/gruell/PID1921091.pdf
Imaris v9.3	Bitplane	https://imaris.oxinst.com
BIAevaluation	Biacore, GE Healthcare	http://www.biacore.com/lifesciences/index.html
CellProfiler Analyst 2.2.1	CEllprofiler	https://cellprofiler.org/
Prism, version 5	Graphpad Software, USA	https://www.graphpad.com/
Python, version 3.0	Python Software Foundation	https://www.python.org/

**Other**		

Cell culture insert	Millicell	Cat#PICMORG50
Nun delta surface 60mm dishes	Nunc	Cat#150288
Nanofibers 6well plate	SIGMA	Cat#Z759333-1EA
SPR Series S Sensor Chip CM5	GE Healthcare	Cat#29104988

### Lead Contact and Materials Availability

Further information and requests for resources and reagents should be directed to and will be fulfilled by the Lead Contact, Elena Seiradake (elena.seiradake@bioch.ox.ac.uk). We are glad to share plasmids published in this study with reasonable compensation by requestor for its processing and shipping.

### Experimental Model and Subject Details

#### Mouse lines

All mice (C75BL/6 background) were housed with 12:12h light/dark cycle and food/water available *ad libitum*. All experimental procedures involving mice were conducted in accordance with regulations set by the government of Upper Bavaria (License number 55.2-1-54-2532-57-2015). All histological experiments were carried out between E15.5 and E18.5.

#### Primary cultures

Cortical explants from E15.5 embryos were dissected out and placed on 60-mm dishes or nanofibers. Coating was performed using 50 μg/ml of FC or Lphn1 proteins and 20 μg/ml of laminin (surface experiments) or 40 μg/ml of FC or Lphn1 proteins with 100 μg/ml poly-D-lysine and 20 μg/ml of laminin (nanofibers). Neurons were dissociated from cortices of E15.5 embryos and cultured on stripes (same coating as explants) or coverslips coated with 0.5mg/ml Poly-D-Lysine in 24 well-plates. Explants and neurons were cultured for 1-3 days *in vitro* at 37°C, 5% CO_2_ in Neurobasal medium supplemented with B27. Samples were fixed with 4% Paraformaldehyde for 10 min and processed for immunostaining.

##### Cell lines

HEK293T cells (ATCC, cat# CRL-3216) and HEK293S cells (ATCC, cat#CRL-3022) used in this study were cultured in Dulbecco’s Modified Eagle Medium, DMEM, supplemented with 10% FBS, 1% NEAA and 1% L-Glutamine at 37°C and 5% CO_2_. K-562 cells (ATCC, cat# CCL-243) used in this study were cultured in phenol red-free Iscove’s Modified Dulbecco’s Medium, IMDM, supplemented with 10% FBS, 1% NEAA and 1% L-Glutamine at 37°C and 5% CO_2_. Protein expression was induced by transfection with plasmid DNA and polyethylenimine as the transfection reagent.

### Method Details

#### Constructs and cloning

The constructs used in this study (Figures 2A–2C) were cloned into pHLSec (Aricescu et al., 2006) or pCAGIG (Addgene, 11159) variant vectors with relevant tags for expression in cell lines or neurons, respectively. For protein purification, we used pHLSec vectors which also code for a C-terminal 6xHis-tag, for SPR we used a C-terminal Avi-tag, and for visualization on HEK or K-562 cells we used pHLSec variants that add an intracellular mVenus, mRuby, and/or an extracellular Flag, Myc and/or HA tag as indicated. For cloning of Ten2^LT^ (1892N+K1894T) we used forward and reverse primers, ctacgatgataaccgcacattcaccctgaggata and cagctgatcgtaaattatcctcagggtgaatgtgcggttatcatcgtag, respectively. For cloning of Lphn1^TL^ (E39N) we used forward and reverse primers, ctatcctgtgagggttattctatagacc and accctcacaggatagatttcgtctaac, respectively. For cloning of Lphn1^TL2^ (L39A, P51G, D54A, D67A, D72A) we used forward and reverse primers, gccgaccctttccagatggagaatgtgcagtgctacttgcctgacgc and ctggaaagggtcggcagcgcagatcttgtcagctgtgcg, respectively. We also used constructs that were from previous papers, Lphn1^FL^ (Jackson et al., 2015) and FLRT^LF^ (Seiradake et al., 2014). In detail, the forward and reverse primers that were used for cloning Lphn1^FL^ (R292N+R294T) were cctgttcttcaacaaagagaataccacaaacatcgtgaagttcgacctgc and gcaggtcgaacttcacgatgtttgtggtattctctttgttgaagaacagg, respectively. The forward and reverse primers used for cloning FLRT^LF^ (R186N+D188T) were gggcttcctgtagacttgcaagagctgaatgtgactgaaaaccgaattgccgtcatatc and gatatgacggcaattcggttttcagtcacattcagctcttgcaagtctacaggaagccc, respectively.

#### Protein expression and crystallization

Unless indicated otherwise, all purified proteins were expressed in adherent HEK293 cells following established procedures (Seiradake et al., 2015). More specifically, for each protein preparation, three liters of HEK cell culture were grown in Dulbecco’s Modified Eagle Medium, DMEM (Life Technologies, cat#21969035), supplemented with 10% FBS (Life Technologies, cat# 10270-106), 1% NEAA (Life Technologies, cat# 11140035) and 1% L-Glutamine (Life Technologies, cat#25030-024) at 37°C and 5% CO_2_. K-562 cells (ATCC, cat# CCL-243). Prior to transfection, the FBS content was reduced to 2%. The cells were transfected with 6 mg of purified plasmid DNA, previously mixed in a 1:2 (mass) ratio of DNA: polyethyleneimine (cat# 208727, Sigma Aldrich) as transfection reagent. The medium was harvested 5-10 days later, buffer-exchanged to 1x PBS (Sigma) supplemented with 150 mM NaCl and 20 mM Tris, pH 7.5. The proteins were purified by loading onto a 5ml HisTrap HP column (GE Healthcare). The column was then washed with 30 column volumes of buffer containing 40 mM imidazole, and the protein eluted using buffer containing 500 mM imidazole. The proteins were then purified using a Superdex200 size exclusion column (GE Healthcare) using buffers containing 150 mM NaCl and 20 mM Tris, pH 7.5. The proteins used for crystallization were expressed under conditions that reduce glycan heterogeneity. Ten2 and Lphn1 (Lec-Olf) proteins were expressed in HEK293T cells in the presence of kifunensine, and Lphn2 Lec was expressed in HEK293S GlnTI^-^ cells (Reeves et al., 2002, Seiradake et al., 2015). Prior to crystallization, the relevant proteins were mixed together in a 1:1 molar ratio, both complexes were concentrated to 4.9 mg/ml and mixed in 1:1 volume ratio with crystallization solution. The Ten2 and Lphn2 Lec complex was mixed with 0.1 M calcium acetate, 0.1 M sodium acetate, pH 4.5, 10% (w/v) PEG 4000, and crystals grew in sitting drops at 4°C before flash-freezing in liquid nitrogen in reservoir solution supplemented with 25% glycerol. The Ten2 and Lphn1 (Lec-Olf) complex was mixed with 0.1 M potassium chloride, 0.1M HEPES pH7.5, 15% (w/v) PEG6000, and crystals grew in sitting drops at 18°C before flash freezing in reservoir solution supplemented with 25% glycerol.

#### Crystallographic analysis

We performed the X-ray diffraction data collection at the Diamond Light Source beamline I03, in each case from a single crystal at 100 K. Data from the Ten2-Lphn2 complex crystals were integrated data up to 3.6 Å resolution using xia2 (Winter et al., 2013) and we solved the structure by molecular replacement using phaser (McCoy et al., 2007) with mouse Lphn3 Lec (PDB ID 5AFB; Jackson et al., 2015) and chicken Ten2 (PDB ID 6FB3; Jackson et al., 2018) as inputs. We placed two copies of each Teneurin and Latrophilin in the asymmetric unit of the P1 cell. After placing Lphn3 Lec, we substituted this with a mouse Lphn2 Lec domain homology model, generated by SWISS-MODEL (Bordoli et al., 2009). Glycans and selected solvent atoms that were present in the high-resolution molecular replacement models were included as appropriate. Given the higher resolution data used to refine the input models of our molecular replacement job, we targeted the model to these structures during refinement. We also used automatic non-crystallographic symmetry restraints, and TLS refinement. Data for the Ten2-Lphn1 complex was integrated to 4 Å resolution using xia2 (Winter et al., 2013) and we solved the structure by molecular replacement using phaser (McCoy et al., 2007) with the Ten2-Lphn2 structure presented here, and a homology model of the Lphn1 Olf domain, generated by SWISS-MODEL (Bordoli et al., 2009). Again, two copies of the complex were found in the asymmetric unit. We refined both structures using programmes in ccp4 (Winn et al., 2011), buster (Smart et al., 2012) and coot (Emsley and Cowtan, 2004). Validation of the models was performed in molprobity (Davis et al., 2007). We created figures using pymol. Sequence conservation analysis was performed with Consurf (Glaser et al., 2003). For this analysis, sequences of all homologs were aligned from *Mus musculus*, *Xenopus tropicalis*, *Gallus gallus* and *Danio rerio* using Clustal Omega (Madeira et al., 2019). Crystallographic details are summarized in Table S1.

#### MD simulation analysis

We followed the same protocol as previously described to refine the interaction surfaces of X-ray crystallography-derived complexes (Jackson et al., 2016). In detail, Molecular dynamics simulations were performed using GROMACS 2018 (Abraham et al., 2015) with an AMBER99SB force field (DePaul et al., 2010, Lindorff-Larsen et al., 2010, Sorin and Pande, 2005). We embedded the complex model in a box of water (SPC/E water model; Berendsen et al., 1987). Na and Cl ions were added up to 150 mM. Energy minimization was performed using the steepest descent algorithm and each system was equilibrated with a constant temperature (canonical ensemble, NVT, 310 K) ensemble for 100 picoseconds (ps), followed by a 100 ps equilibration at constant pressure (isothermal- isobaric, NPT, 1 bar). For equilibration and production runs, we applied the velocity-rescaling thermostat (Bussi et al., 2007) on protein and solvent, coupled with the Parrinello–Rahman barostat (Parrinello and Rahman, 1981), with a time constant of 2.0 ps and compressibility of 4.5x10^−5^ bar^-1^. Long-range electrostatics were modeled using the Particle-Mesh Ewald method (Darden et al., 1993, Essmann et al., 1995). All bonds were treated using the LINCS algorithm (Hess, 2007). The integration time step was 2 femtoseconds. We performed two different simulations: (i) 500 ns of unrestrained simulation to assess the stability of the Ten2-Lphn2 Lec complex; and (ii) 50 ns semi-restrained simulation. For the latter, the Teneurin backbone was constrained and we applied distance restraints on the Latrophilin backbone. By doing this we allowed Latrophilin to move with respect to Teneurin while keeping the backbone of both proteins in the configuration determined by X-ray crystallography. Side chains were allowed to move freely in this simulation. The restrained simulation was used to analyze H-bonds formed at the protein interfaces throughout the last 40 ns of simulation using the MDAnanlysis library (Michaud-Agrawal et al., 2011) (relevant Jupyter Notebook and tcl script available at: https://github.com/MChavent/Hbond-analysis). We used a donor-acceptor distance cut-off of 3.0 Å and a cut-off angle of 120°. The stability was defined as correlating with the percentage of the simulated time in which an atom forms the H-bond with its partner

#### Surface Plasmon Resonance

Equilibrium binding experiments were performed at 25°C using a Biacore T200 instrument (GE Healthcare) using PBS + 0.005% (v/v) polysorbate 20 (pH 7.5) as running buffer. The regeneration buffer was 2 M MgCl_2_. Latrophilin constructs were biotinylated enzymatically at a C-terminal Avi-Tag and coupled to a streptavidin-coated CM5 chip (GE healthcare, cat# 29104988). Data were analyzed using the BIAevaluation software (Biacore, GE Healthcare). Indicative K_D_ and R_max_ values were obtained by nonlinear curve fitting of a 1:1 Langmuir interaction model (bound = R_max_/(K_D_ +C), where C is analyte concentration calculated as monomer (Table S2).

#### Cell surface expression tests

HEK293 cells were transiently transfected with transmembrane constructs of Teneurin or Latrophilin that were fused to an intracellular mVenus-tag and an extracellular HA, Flag or Myc tag. Depending on the tag included, anti-HA (SIGMA-Aldrich, Cat No. H3663), ant-Flag (SIGMA-Aldrich, Cat No. F1804) or anti-Myc (Abcam, Cat No. ab19233) were added to the cells at a concentration of 5 μg/ml. Cells were incubated for 1 hour on ice and washed with phosphate buffer saline (PBS, Lonza). Cells were fixed in 4% PFA (20min, on ice), immunostained with Cy3-labeled anti-mouse antibody (Abcam, Cat No. ab97035) or anti-chicken A568 antibody (Abcam, Cat No. ab175711) and mounted. Non-transfected cells acted as negative control.

#### Cell-based binding assays

HEK293 cells were transiently transfected with the same constructs described above. His-tagged purified proteins were pre-clustered with 6x-His Tag antibody (Life Technologies, Cat No. 372900) for 1hr on ice and were added to the cells at a concentration of 5 or 10 μg/ml, for purified Lphn or Ten2 ectodomain samples, respectively. Cells were washed with phosphate buffer saline (PBS) and fixed in 4% PFA (20min, on ice), immunostained with Cy3-labeled anti-mouse antibody (Abcam, Cat No. ab97035) or anti-chicken A568 antibody (Abcam, Cat No. ab175711) and mounted. Images were analyzed using ImageJ. For each image, the integrated density (the sum of the values of the pixels in the image) corresponding to bound protein was quantified (red) and divided by the integrated density corresponding to mVenus overexpression (green).

In case of experiments where HEK293 expressing FLRT3 were incubated with purified Lphn1 (Lec-Olf) or Ten2 ectodomain. HEK293 cells were transfected with a pcDNA3 vector (Invitrogen) containing full-length mouse FLRT3 with a C-terminal FLAG-tag. 100 nM Ten2 or Lphn1 (Lec-Olf) were pre-clustered with 50 nM Anti-6XHis-tag FITC (Abcam, ab3554) or Anti-6XHis-tag DyLight 650 (Abcam, ab117504) antibodies for 1hr at room temperature and were added to the cells simultaneously. Following an incubation for 20 min at room temperature, cells were washed with PBS. Cells were then fixed in 4% PFA for 10 min at room temperature, permeabilized in PBS containing 0.1% Triton (CarlRoth) and 1% bovine serum albumin (Sigma) and immunostained for FLRT3 overexpression with an anti-FLAG antibody (1:1000, Sigma, F9291).

#### Cell aggregation assays

K-562 cells were cultured according to the manufacturer’s instructions using Iscove’s Modified Dulbecco’s Medium without phenol red supplemented with 10% FBS. Cells were transfected with pHLSec plasmids coding for Teneurin (N-terminal mVenus) or Lphn (C-terminal mRuby) as indicated, using Lipofectamine LTX with PLUS reagent (Life Technologies, 15338100). 24 hours after transfection, the two population of cells were mixed in 24-well plates and incubated at 37°C, 5% CO_2_ and 250 rpm for ∼30 min.

#### Stripe assays

50 μg/ml His-tagged protein was mixed with 120 μg/ml Cy3-conjugated αFc (Life Technologies A11014) in PBS. Matrices (90 μm width) (Knöll et al., 2007) were placed on 60 mm dishes and proteins injected. After 30 min incubation at 37°C, dishes were washed with PBS and matrices removed. Dishes were coated with 50 μg/ml Fc protein mixed with 120 μg/ml anti-hFc (Jackson ImmunoResearch 109-005-098) for 30 min at 37°C and washed with PBS. Stripes were further coated with 20 μg/ml Laminin in PBS for at least 2 hours and washed with PBS. Cortical neurons (E15.5) or explants (E15.5) were cultured on the stripes in Neurobasal medium supplemented with B27 (Invitrogen) and, in case of explants, 0.4% methyl-cellulose (Sigma). After 24 (neurons) or 48 (explants) hours and fixed with 4% PFA in PBS for 20 min at room temperature (RT). Neurons and explants were washed and incubated with rabbit monoclonal anti-beta-III tubulin antibody (Sigma) after 20 min permeabilization in 1% BSA, 0.1% Triton X-100/PBS. Cy2 anti-rabbit IgG secondary antibody (Jackson ImmunoResearch, cat#111-225-144) was used to visualize the tubulin signal. Nuclei were counterstained with DAPI before mounting. The numbers of beta-III-tubulin+ or DAPI+ pixels on red or black stripes were quantified using ImageJ (version 1.51p) (Schneider et al., 2012).

#### In Utero Electroporation

In utero electroporation (IUE) was performed at E13.5 or E15.5 on anesthetized C57BL/6 mice, as decribed below. DNA plasmids were used at 2 μg/μl and mixed with 1% fast green (Sigma, final concentration 0.2%). Knockdown of Ten2 by shRNA used the following sequences embedded in pCAG-miR30 plasmid: shRNA1: CTCCCTGTACGTTCTGGAGAAC, shRNA2: TTCACATACAGCGCTGACAATG. CRISPR sequences embedded in pX458 plasmids were: CRISPR#1: AGCTCCTGCGCCCAACGACC CRISPR#2: AACAGTAACCACTTCGGTGT. Scrambled sequence as control was: GCTCGCACACTAGCCGCCAC. Plasmids were injected into the ventricle with a pump controlled micropipette. After injection, six 50 ms electric pulses were generated with electrodes confronting the uterus above the ventricle. The abdominal wall and skin were sewn and the mice were kept until the desired embryonic stage. The pCAG-miR30 plasmids were validated in HEK293T cells, by co-transfection with mouse Ten2 (ecto) construct or chicken Ten2 (ecto), which acted as a non-sensitive control. The expression of Ten2 was analyzed by western blotting, using mouse anti-His antibody (QIAGEN, 34660). Secreted versions of Lphn1(Lec-Olf) and Lphn1 (Lec-Olf) ^TL-FL^ used in IUE experiments were cloned into the pCAGIG vector, and encoded with a C-terminal Myc tag. The expression of these constructs were validated by expression in HEK293T cells and analyzed on western blots using chicken anti-Myc antibody (Abcam, ab19233).

#### Brain slice time-lapse experiments

Embryos were electroporated at E15.5 as described above using pCAG-mCherry (control neurons) together with pCAG-Ten2-IRES-GFP or pCAG-Ten2LT-IRES-GFP. After 48 hs, embryonic brains were dissected in ice cold sterile filtered and aerated (95% O_2_/5% CO_2_) dissection medium (15.6 g/l DMEM/F12 (Sigma); 1.2 g/l NaHCO3; 2.9 g/l glucose (Sigma); 1% (v/v) penicillin streptomycin (GIBCO, cat#15140148)). Brains were embedded in 4% low melting agarose (Biozym, cat#840101) and cut into 300μm thick sections using a vibratome (Leica, VT1200S). Sections were suspended in a collagen mix (64% (v/v) cell matrix type I-A, Nitta Gelatin; 24% (v/v) 5 x DMEM/F12; 12% (v/v) reconstitution buffer (200mM HEPES; 50mM NaOH; 260mM NaHCO_3_) and transferred onto a cell culture insert (Millicell; PICMORG50). Sections were incubated for 10 min at 37°C to solidify collagen. 1.5 mL slice medium (88% (v/v) dissection medium; 5% (v/v) horse serum; 5% (v/v) fetal calf serum; 2% (v/v) B27 supplement (GIBCO, cat#17504044); 1% (v/v) N-2 supplement (GIBCO, cat#17502001)) was added into the dish surrounding the culture insert and incubated for 30 min at 37°C. Before start of time-lapse experiment, culture medium was added on top of the sections to allow objective immersion. Sections were imaged using a 20x water immersion objective on a Leica SP8 confocal microscope system equipped with a temperature-controlled carbon dioxide incubation chamber set to 37°C, 95% humidity and 5% CO_2_. Sequential images were acquired every 20 min for 14-60 hr. Single cell movement was tracked using the Fiji plugin “Manual Tracking.” Single cell track analysis and plotting was carried out using homemade python scripts.

#### Pull-down experiments

Fresh E15.5 mouse cortices were homogenized for 1min at 4°C with an electric homogenizer using the following lysis buffer: 50 mM Tris-HCL (pH 7.4), 150mM NaCl, 2mM EDTA, 1% Triton X-100 and protease inhibitors (Roche ref. 04693116001). Samples were incubated on ice for 20 min and centrifuged for 10 min at 3000 rpm. Supernatant was collected and protein was measured using the Bio-Rad protein assay (Biorad, 5000001). 1 mg of protein at a final concentration of 2 μg/μl in lysis buffer (volume: 500 μl) was used for each pull-down. Control pull-down contained lysate and 2 μg of goat anti-human IgG antibody (Jackson Immunoresearch, 109-005-098) while FLRT3 pull-down used 2 μg of goat anti-human FLRT3 antibody (R&D, AF2795). Samples were incubated overnight at 4°C under rotatory agitation. The next day, 10 μg of Sepharose beads were added to each sample (Amersham CL-4B, 17-01780-01, 50% v/v in lysis buffer) and incubated for 4 hours under rotatory agitation. Sepharose beads were centrifuged for 5 min at 3000 rpm and washed three times (first wash with 400 μl of lysis buffer, second wash with 1:1 (v/v) lysis buffer:PBS, last wash only PBS). Pulled-down samples were processed for mass spectrometry (MaxQuant run, Proteomic facility, Max Planck Institute of Biochemistry, Martinsried, Germany) or standard western blot. For western blotting, samples were run on 6% SDS gels, transferred to PVDF membranes and blotted for Ten2 (Novus Biologicals, rabbit, NBP2-55763) or FLRT3 (R&D, AF2795). For mVenus-tagged Ten2 pull-downs, adherent HEK293-T cells were transfected for 24 hours, with 0.5 μg pHLSec DNA coding for murine FLRT2 (Flag tagged) and 3 μg of pHLSec DNA coding for chicken Ten2 (mVenus tagged), per 2 mL of cell culture. Separately, cells were transfected with 3 μg of murine Lphn3, per 2 mL cell culture. 4 mL of each cell was used per experiment. Next day, cells transfected with Lphn3 were washed with PBS and incubated at 37°C with PBS supplemented with 1mM EDTA for 5min. The PBS/EDTA solution was then removed and cells were resuspended in 1ml DMEM media supplemented with 10% FBS, and pelleted at 100 x g for 4 min. Equal volumes of Lphn3-cells were added to adherent cells transfected with Ten2/FLRT2 and incubated at 37°C for 1hr. Cells were resuspended in ice-cold lysis buffer (1% Triton X-100, 50 mM Tris-HCl at pH7.5, 150mM NaCl and EDTA-free protease inhibitors (Sigma)). Cells were disrupted by mechanical force, and incubated on ice for 20 min. Cell debris was removed by centrifugation and expression control samples were taken at this point. The lysate was incubated with 2 μg/ml of rabbit anti-GFP antibody (Life Technologies, A11122) at 4°C for 1 hour under rotary agitation. 45 μl of a 50% suspension of pre-blocked Protein-G Sepharose 4 FF (Sigma, cat#P3296) was added per 1 mL of lysate, and incubated for 2 hours. After incubation, Sepharose beads were pelleted and washed twice with ice-cold lysis buffer, once with 1:1 ice-cold lysis buffer:PBS, once with ice-cold PBS, then resuspended in PBS and added with SDS-loading buffer. FLRT protein was detected using anti-Flag (Sigma, F1804) on a western blot. Band intensities were analyzed using ImageJ (Fiji) and normalized within each blot.

#### Explant culture on nanofibers

6 well-plate parallel nanofibers (700nm width, Sigma, Z759333-1EA) were coated with 40 μg/ml of specified protein (FC, Lphn1(Lec-Olf) and its mutant versions) and 100 μg/ml poly-D-lysine (Sigma) in PBS overnight (37°C, 65% humidity and 5% CO2). Next day, plates were washed with PBS and coated with 20 μg/ml laminin in PBS overnight. The next day, plates were washed with PBS and 36 cortical explants (E15.5) were placed per well (6x6 grid, see Figure S5E) and cultured for 2 days in Neurobasal medium supplemented with B27 (Invitrogen) supplemented with 0.4% methyl-cellulose (Sigma). Then the explants were fixed with 4% PFA for 20min, washed with PBS and incubated with rabbit monoclonal anti-beta-III tubulin antibody (Sigma) after 20 min of permeabilization in 1% BSA, 0.1% Triton X-100/PBS. Cy2 anti-rabbit IgG secondary antibody (Jackson ImmunoResearch, Cat#111-225-144) was used to visualize the tubulin signal. Nuclei were counterstained with DAPI before mounting. Mosaic images of each well were taken with SP8 (Leica) microscope. Analysis of the distance covered by cells labeled with DAPI and axons labeled with beta-III-tubulin was done in a semi-automatic mode using a custom ImageJ macro.

#### Cultured neuron time-lapse experiments

To perform the time-lapse analysis of cortical neurons migrating on surfaces coated with FC (control), murine Lphn1 (Lec-Olf) or Lphn1 (Lec-Olf) ^TL-FL^, cortical explants from E15.5 mouse embryos were cultured on 60-mm dishes that were coated for 30 min with 50 μg/ml of each protein in PBS. After 30 min, the dish was washed with PBS and coated with 20 μg/ml Laminin in PBS for at least 2 hours. The dish was next washed with PBS and cortical explants (E15.5) were cultured. After 2 hours in culture, samples were imaged with a Zeiss Axiovert 200M microscope equipped with a temperature-controlled carbon dioxide incubation chamber set to 37°C, 65% humidity and 5% CO_2_. Illumination was provided by an X-Cite lamp (series 120, Lumen Dynamics Group), and images were recorded by a Coolsnap HQ camera (Photometrics). Sequential images were acquired every 10 min. Analysis and tracking of all the neurons exiting the explant was carried out in Imaris (Bitplane) using the automatic tracking module.

For time-lapse analysis of GFP+ neurons and axons challenged with alternate Lphn1(Lec-Olf) or Lphn1 (Lec-Olf) ^TL-FL^ stripes, E13.5 mouse embryos were electroporated with PCAGIG plasmid and cortical explants (E15.5) were placed on stripes as described above. After 2 hours in culture, cortical explants were imaged with a Zeiss Axiovert 200M microscope equipped with a temperature-controlled incubation chamber as described above. Sequential images were acquired every 10 min. Analysis was carried out using ImageJ, all contact events taking place in less than 2 frames were considered as repulsive events.

Time-lapse experiments of GFP+ neurons on nanofibers started after 2 hours of culturing E15.5 cortical explants, that had previously been electroporated at E13.5. Samples were imaged with a Zeiss Axiovert 200M microscope equipped with a temperature-controlled incubation chamber as described above. Sequential images were acquired every 10 min.

#### Surface staining and co-localization on cortical neurons

Cortical neurons (E15.5) were cultured on coverslips (13mm diameter, 24 well-plate, #1.5) coated with poly-D-lysine (Sigma, 0.5mg/ml) in Neurobasal medium (without phenol red for super resolution microscopy) supplemented with B27 (GIBCO Cat#17504044). After 2DIV, 0.2μg of Lphn1(Lec-Olf) or double mutant (Lec-Olf)^TL-FL^ protein clustered with anti-his Alexa 488 antibody (Thermofisher cat#MA1-21315-A488, ratio 4:1 protein:Ig) was added to cortical neurons for 20 min at room temperature (RT). Neurons were washed twice with PBS and incubated with pre-clustered FLRT3 antibody (goat Ig, R&D, AF2795) with anti-goat Alexa-594 (Jackson, ImmunoResearch, Cat#705-585-003, ratio 4:1) and N-terminal Ten2 antibody (sheep, R&D, AF4578) with anti-sheep Alexa-647 (Jackson, ratio 4:1) for 20 min at RT. Neurons where washed twice with PBS and fixed for 20 min with 4% PFA at RT. Neurons were washed with ammonium chloride in PBS (50mM) for 5 min to quench the excess of PFA, followed by two washes with PBS and mounted with Immu-mount (Thermofisher, cat#10622689) for super resolution. In case of confocal microscopy, nuclei were stained with DAPI and samples were mounted with Dako (Agilent, cat#S3023). Confocal acquisition was done using a SP8 laser scanning confocal spectral microscope (Leica Microsystems Heidelberg). Images were taken using a 63 × objective with a 2 × digital zoom and 1 Airy disk pinhole. Co-localization was quantified using the co-localization plugin of Image (version 1.51p) (Schneider et al., 2012).

#### Super-resolution imaging

Neurons were prepared as described above, and mounted in Immu-Mount (Thermo Fisher Scientific) for Single Molecule Localization Microscopy (SMLM) imaging on a Leica SR GSD system. The following settings where used for SMLM data acquisition: 642 nm laser at 40% of 500 mW output power for Alexa647, 560 nm laser at 100% of 500 mW output power for Alexa594 and 488 nm laser at 70% of 300 mW output power for Alexa488. For Alexa488 and Alexa647 low to moderate laser intensities of 405 nm were used for “back-pumping” to enhance single molecule blinking. For the position determination of the single fluorescent molecules we used a maximum-likelihood-based algorithm with a sliding window for background subtraction (Grull et al., 2011). SMLM images where reconstructed based on the localization accuracies of the individual single molecule positions.

#### RNaseq analysis

RNaseq data were accessed from the published NCBI Gene Expression Omnibus with accession numbers GEO:GSE10881 (Kawaguchi et al., 2008) and GSE65000 (Florio et al., 2015). Values for specific genes from individual cells were averaged and displayed as whisker plots.

#### RNA *In Situ* Hybridization (ISH) and Immunohistochemistry

Embryonic brains were fixed in 4% PFA over-night and sectioned. 10 μm Cryo-sections were pre-treated using the RNAscope Universal Pretreatment Kit (Advanced Cell Diagnostics, Cat#322380). RNA *In Situ* Hybridizations (ISH) were performed using the RNAscope Fluorescent Multiplex Reagent Kit (Advanced Cell Diagnostics, Cat#320850) according to manufacturer’s instructions. The target genes (mouse Lphn1-3 and Ten1-4) are also listed in the Key Resources Table. Following ISH, sections were immunostained using mouse anti-Pvim 1/300 (Abcam, Cat#ab20346) and rat anti-Ctip2 1/600 (Abcam, Cat#ab123449) in combination with the Alexa Fluor 488 secondary antibodies 1/200 (Jackson Immunoresearch). Images were acquired using a Leica TCS SP8 confocal laser scanning microscope and processed with ImageJ software.

### Quantification and Statistical Analysis

Statistical analyses of were performed using GraphPad Prism, employing a two-tailed unpaired Student’s t test when comparing two groups (Figures S2G, 3C, 3F, 3I, S3H, S3L, S3P, and S3R) or one-way ANOVA test with Tukey’s post hoc analysis when comparing multiple groups (Figures 2E, 2F, 2I, 4B, 4H, S4B, S4D, 5B, 6D, 6F, 6G, S6E, 7C, 7E, 7G, S7B, and S7D) where P values represent ^∗^p ≤ 0.05, ^∗∗^p ≤ 0.01, ^∗∗∗^p ≤ 0.001 and ^∗∗∗∗^p ≤ 0.0001. All data are presented as the mean ± s.e.m, whisker plots or dot plots. All sample sizes and definitions are provided in the figure legends.

### Data and Code Availability

The crystallography data and models generated during this study are available for download in the Protein Data Bank (PDB accession numbers 6SKE, 6SKA). The code generated to analyze molecular dynamic simulations is freely available from https://github.com/MChavent/Hbond-analysis, or available upon reasonable request.

## Supporting Citations

The following references appear in the Supplemental Information: Brasch et al., 2018, Dionne et al., 2018.
