# Supplementary material for: Structural Basis of Teneurin-Latrophilin Interaction in Repulsive Guidance of Migrating Neurons
Source: Cell. 2020 Jan 23;180(2):323–339.e19. doi: 10.1016/j.cell.2019.12.014 (PMC6978801; doi:10.1016/j.cell.2019.12.014)
Supplement: Document S1. Tables S1 and S2 [file mmc1.pdf]

Cell, Volume *180*

Supplemental Information

Structural Basis of Teneurin-Latrophilin Interaction in Repulsive Guidance of Migrating Neurons

Daniel del Toro, Maria A. Carrasquero-Ordaz, Amy Chu, Tobias Ruff, Meriam Shahin, Verity A. Jackson, Matthieu Chavent, Miguel Berbeira-Santana, Goenuel Seyit-Bremer, Sara Brignani, Rainer Kaufmann, Edward Lowe, Rüdiger Klein, and Elena Seiradake

**Data collection**		
Protein	chicken Ten2 and murine Lphn2 Lec	chicken Ten2 and murine Lphn1 Lec-Olf
PDB code	6SKE	6SKA
Space group	P1	$P4_322$
Cell dimensions		
a, b, c (Å)<br>α, β, γ (°)	90.87 109.92 152.21<br>90.19 93.99 111.93	96.26 96.26 809.22<br>90.0 90.0 90.0
Resolution (Å)	84-3.6 (3.71-3.6)	202-3.86 (4.05-3.86)
R_{meas}	0.334 (212)	0.556 (3.46)
I/σ(I)	3.47 (0.56)	6.19 (1.41)
Highest resolution shell with I/σ(I) > 2	4.49 - 4.33 (R_{meas} of shell = 0.532)	4.31-4.22 (R_{meas} of shell = 1.87)
$CC_{1/2}$	0.97 (0.25)	0.99 (0.45)
Completeness (%)	96 (96)	91.2 (36.2)
Redundancy	1.8 (1.8)	12.8 (13.7)
**Refinement**		
Resolution (Å)	84-3.6 (3.64-3.6)	52-3.86 (3.98-3.86)
R_{work}/R_{free*}	0.27/0.27 (0.28/0.28)	0.26/0.26 (0.25/0.28)
No of atoms		
protein	29198	17313
other	580	305
solvent	210	-
mean B-values ($Å^2$)	99.4	134
Ramachandran plot		
favoured (%)	96.5	95.21
outliers (%)	0.24	0.64
Bond length deviations (Å)	0.007	0.007
Bond angle deviations (°)	0.97	0.95

Table S1 related to Figure 1. Crystallographic statistics for deposited structures 6SKE and 6SKA.

Ligand - Analyte	Apparent K_D	RU analyte immobilised	Bmax	R^2
mLphn3^{ecto} - mTen2^{ecto}	490 nM	730	874	0.995
mLphn2^{ecto} - mTen2^{ecto}	100 nM	700	750	0.971
mLphn1^{ecto} - mTen2^{ecto}	35 nM	670	1014	0.865
mLphn3^{ecto} - gTen2^{ecto}	680 nM	730	1173	0.991
mLphn2^{ecto} - gTen2^{ecto}	60 nM	700	985	0.931
mLphn1^{ecto} - gTen2^{ecto}	40 nM	670	1160	0.872
mLphn1$^{Lec-Olf}$ - mTen2^{ecto}	290 nM	220	473	0.991
mLphn1$^{Lec-Olf}$ - gTen2^{ecto}	590 nM	220	631	0.999

Table S2 related to Figure 2. Apparent binding affinities calculated from SPR measurements using Latrophilin and Teneurin constructs are shown. Here we use the following nomenclature for brevity: gTen2 = chicken Ten2, mTen2= murine Ten2, mLphn = murine Lphn. The construct boundaries and mutant types are indicated in superscript. K_D and R_{max} values were obtained by nonlinear curve fitting of a 1:1 Langmuir interaction model (bound = R_{max}/(K_D +C), where C is analyte concentration calculated as a monomer. Note that Teneurin protein is dimeric and so these values are indicative only. All Ten^{ecto} proteins were injected at the same molar concentrations. As done previously (Brasch et al., 2018; Dionne et al., 2018), the comparison of the resulting raw binding curves (Fig. 2G-H and S2C-E) is a more reliable way to assess relative binding.
